# Temporal Trends and Demographic Disparities in Mortality Due to Coronary Artery Disease and Hypertension in Adults Aged 25 and Above in the United States (2000 to 2024)

**DOI:** 10.1002/clc.70465

**Published:** 2026-09-04

**Authors:** Muhammad Shayan Tariq, Rai Muhammad Umar Khan, Asad Ullah Farooq, Kinza Raza, Maryam Athar, Shumail Tariq, Muhammad Raza Asif, Noor Ul Huda Ramzan, Ameer Haider Cheema

**Affiliations:** ^1^ Department of Medicine Faisalabad Medical University Faisalabad Pakistan; ^2^ Department of Medicine King Edward Medical University Lahore Pakistan; ^3^ Department of Medicine Bakhtawar Amin Medical & Dental College Multan Pakistan; ^4^ Department of Medicine Dow University of Health Sciences Karachi Pakistan; ^5^ Department of Medicine Shalamar Medical and Dental College Lahore Pakistan; ^6^ Department of Medicine Allegheny General Hospital Pittsburgh Pennsylvania USA; ^7^ Department of Medicine University of Texas SouthWestern Dallas Texas USA

**Keywords:** cardiovascular disease, CDC WONDER, coronary artery disease, health disparities, hypertension, mortality trends

## Abstract

**Introduction:**

Coronary artery disease (CAD) remains a leading cause of mortality, and hypertension serves as a primary, modifiable risk factor. While cardiovascular mortality historically declined, recent data indicate this trajectory has plateaued and begun to reverse. This study analyzes the temporal trends and demographic disparities in mortality due to CAD and hypertension among adults in the United States.

**Methods:**

This retrospective study utilized the CDC WONDER database to extract mortality data from 2000 to 2024. Deaths among adults aged 25 years and older with CAD and hypertension were identified. Age‐adjusted mortality rates (AAMRs) per 100 000 population were calculated. Temporal patterns and annual percent change (APC) were evaluated using Joinpoint regression analysis.

**Results:**

Between 2000 and 2024, 3 776 396 deaths occurred due to CAD and hypertension among US adults. Overall AAMR rose from 57.22 to 76.95, driven by a steep 2018–2021 increase (APC: 10.13%) before a recent decline (2021–2024, APC: −3.50%). By 2024, male AAMR (106.43) doubled female AAMR (52.68). Non‐Hispanic African American adults experienced the highest AAMR (96.90). Non‐metropolitan areas saw greater sustained increases (AAPC: 2.79%) than metropolitan areas (AAPC: 1.23%), with the South recording the highest regional AAMR (70.25; AAPC: 1.53%). While essential hypertension mortality stabilized, hypertensive heart disease (AAPC: 4.35%) and renal disease (AAPC: 8.83%) surged. Chronic ischemic heart disease remained the leading CAD subtype (AAPC: 1.37%).

**Conclusion:**

CAD and hypertension‐related mortality among adults has increased significantly over the past 25 years, exposing significant demographic disparities. Targeted public health strategies prioritizing optimal blood pressure management and equitable healthcare access are essential to mitigate this escalating burden.

## Graphical abstract

CAD‐related mortality among hypertensive adults has increased substantially over the past 25 years, especially among males and Non‐Hispanic African American adults, with a concerning upward trend in the younger population exposing significant demographic disparities. Targeted strategies prioritizing optimal blood pressure management and equitable healthcare access are essential to mitigate this escalating burden.

## 1 Introduction

Coronary artery disease (CAD) remains the leading cause of mortality in the United States, accounting for approximately one in every four deaths [1]. Hypertension, affecting nearly half of the US adult population, serves as a primary, modifiable risk factor for the development and progression of CAD [2]. The synergistic relationship between elevated blood pressure and coronary atherosclerosis accelerates end‐organ damage, leading to higher rates of myocardial infarction, heart failure, and premature death [3].

Historically, public health initiatives and advances in pharmacotherapy (e.g., statins and antihypertensives) drove a significant decline in CVD mortality during the late 20th and early 21st centuries. Between 1999 and 2010, the age‐adjusted mortality rate (AAMR) for ischemic heart disease and related cardiovascular conditions saw a steady reduction [4]. However, recent epidemiological data indicate a concerning shift in this trajectory. Since 2011, the rate of decline has plateaued, and in certain demographic subgroups, mortality rates have begun to reverse and rise [5, 6]. This reversal has been attributed to the rising prevalence of obesity, diabetes mellitus, and suboptimal control of hypertension across the population [7].

Furthermore, the onset of the COVID‐19 pandemic in 2020 exacerbated these trends, resulting in a sharp spike in cardiovascular mortality likely driven by both direct viral mechanisms and indirect disruptions to healthcare access [8]. Although previous studies have examined overall cardiovascular or general CAD mortality trends, they often fail to distinguish the distinct trajectory of CAD mortality specifically occurring in adults with hypertension. This represents a critical knowledge gap, as understanding the demographic and geographic disparities unique to this high‐risk population is essential for guiding targeted interventions.

This study utilizes death certificate data from the Centers for Disease Control and Prevention (CDC) Wide‐ranging Online Data for Epidemiologic Research (WONDER) database to analyze the temporal trends in mortality attributed to co‐occurring CAD and hypertension in the United States from 2000 to 2024. By examining these trends across age, sex, race/ethnicity, and geographic regions, this analysis aims to identify high‐risk populations and quantify the impact of the recent reversal in CAD mortality improvements.

## 2 Materials and Methods

### 2.1 Study Setting and Population

This retrospective population‐based mortality study utilized data from the CDC Wide‐Ranging Online Data for Epidemiologic Research (CDC WONDER) Multiple Cause of Death database from January 1, 1999, through December 31, 2024 [9].

The analysis identified death certificates on which both CAD and hypertension were listed among the causes of death. Both CAD and hypertension could be recorded as either the underlying or a contributing cause of death. CAD‐related deaths were identified by the International Classification of Diseases, Tenth Revision (ICD‐10) using codes I20−I25, while hypertension was identified using codes I10−I15, which have been previously validated for identifying these conditions in administrative datasets [10, 11]. The analysis was restricted to adults aged 25 years and older. Because the dataset is publicly accessible and deidentified, ethical approval was not required. To ensure comprehensive and transparent reporting, we followed the Strengthening the Reporting of Observational Studies in Epidemiology (STROBE) guidelines [12].

### 2.2 Data Extraction

Three authors collected and verified the data: two extracted the information independently, and a third verified its accuracy. Key variables included year, age, sex, race/ethnicity, and geographic indicators such as state and region. Regions were categorized in accordance with the US Census Bureau classification into Northeast, Midwest, South, and West [13]. The National Center for Health Statistics Urban‐Rural Classification Scheme was used to assign urban and rural classifications [14]. The categories of place of death included homes, hospices, long‐term care or nursing facilities, and medical facilities (inpatient, outpatient, or emergency department).

Race and ethnicity were recorded as documented on death certificates according to the classification standards of the US Office of Management and Budget and included non‐Hispanic (NH) White, NH African American, NH American Indian or Alaska Native, NH Asian or Pacific Islander, and Hispanic or Latino populations. These categories were based on the race and ethnicity classifications available in CDC WONDER. To ensure consistency in race classification throughout the study period (2000–2024), NH Asian and NH Pacific Islander populations were analyzed as a single category. This approach reflects the structure of the CDC WONDER mortality data available throughout the study period and facilitates temporal comparisons despite changes in race reporting after 2020. Since the CDC WONDER provides data for urbanization status until 2020, our results for urbanization were reported till 2020. For age‐based analyses, decedents were categorized into three groups: 25–44 years, 45–64 years, and ≥ 65 years. Mortality rates within these age strata were calculated as age‐specific rather than age‐adjusted because each estimate was restricted to a predefined age group. Additional subgroup analyses were performed based on CAD subtypes (including angina pectoris, acute myocardial infarction, and chronic ischemic heart disease [CIHD]) and hypertension subtypes (primary and secondary), as defined by ICD‐10 coding.

### 2.3 Statistical Analysis

Crude mortality rates and AAMRs per 100 000 population were computed for the study period from 2000 to 2024. The denominator for mortality rates was the corresponding US population aged ≥ 25 years, rather than a population of individuals with hypertension. Age adjustment was performed using the 2000 US standard population [15].

Demographic and geographic variables, including age group, sex, race/ethnicity, state, region, urban‐rural classification, place of death, and disease subtypes, stratified mortality rates. Temporal patterns in mortality were evaluated using the Joinpoint Regression Program (Version 5.2.0), which applies log‐linear regression to identify significant changes in mortality trends [16]. Annual percent change (APC) and average APC (AAPC) were estimated along with 95% confidence intervals (CIs). Confidence intervals for age‐adjusted rates were calculated using the Tiwari modified gamma approach, while Poisson‐based 95% CIs accompanied crude rates. Statistical significance was determined using two‐sided *t*‐tests, with a threshold of *p* ≤ 0.05.

## 3 Results

A total of 3 776 396 deaths attributed to CAD and hypertension in adults aged ≥ 25 years in the United States from 2000 to 2024. Annual deaths increased substantially over the study period, from 102 151 in 2000 to 217 849 in 2024, highlighting the growing burden of CAD and hypertension‐related mortality among adults in the United States (Supporting Information S1: Table 2).

### 3.1 Overall Mortality Trends

**Figure 1:**
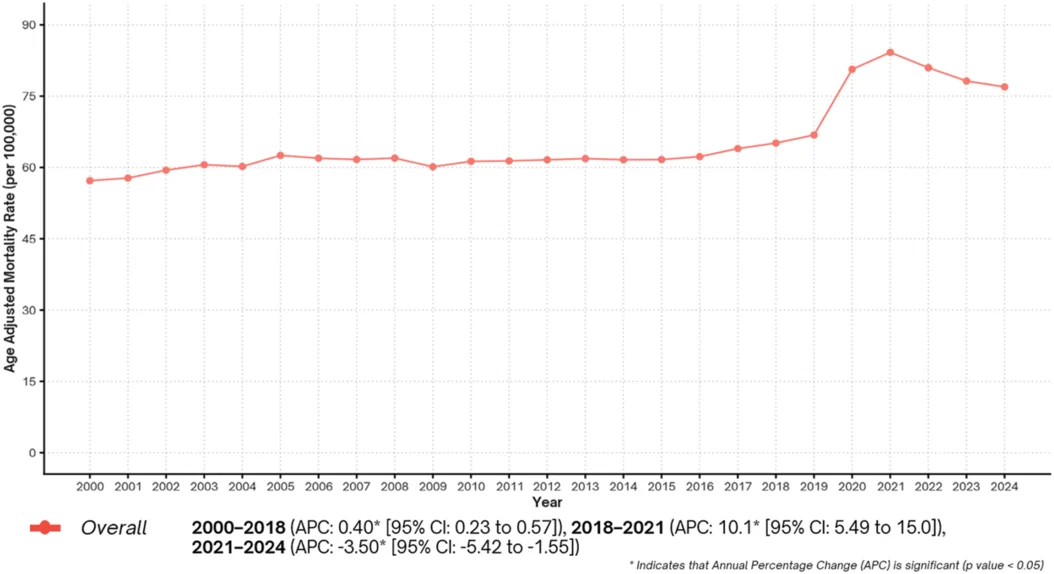
Overall trend in coronary artery disease and hypertension‐related age‐adjusted mortality rates among US adults per 100 000, 2000 to 2024.

The AAMR for CAD and hypertension‐related deaths among adults aged 25 years and older was 57.22 per 100 000 in 2000 and increased to 76.95 per 100 000 in 2024, with an average AAMR of 65.75 per 100,000 during the study period. The AAMR showed a modest but significant increase from 2000 to 2018 (APC: 0.40; 95% CI: 0.23 to 0.57; *p* = 0.000106), followed by a steep and significant increase from 2018 to 2021 (APC: 10.13; 95% CI: 5.49 to 14.97; *p* = 0.000193), and then a significant decline from 2021 to 2024 (APC: −3.50; 95% CI: −5.42 to −1.55; *p* = 0.001564). Overall, the findings demonstrate a marked rise in AAMR through 2021, followed by a decline in the subsequent years. (Figure 1) (Supporting Information S1: Table 3).

### 3.2 Mortality Trends Stratified by Gender

**Figure 2:**
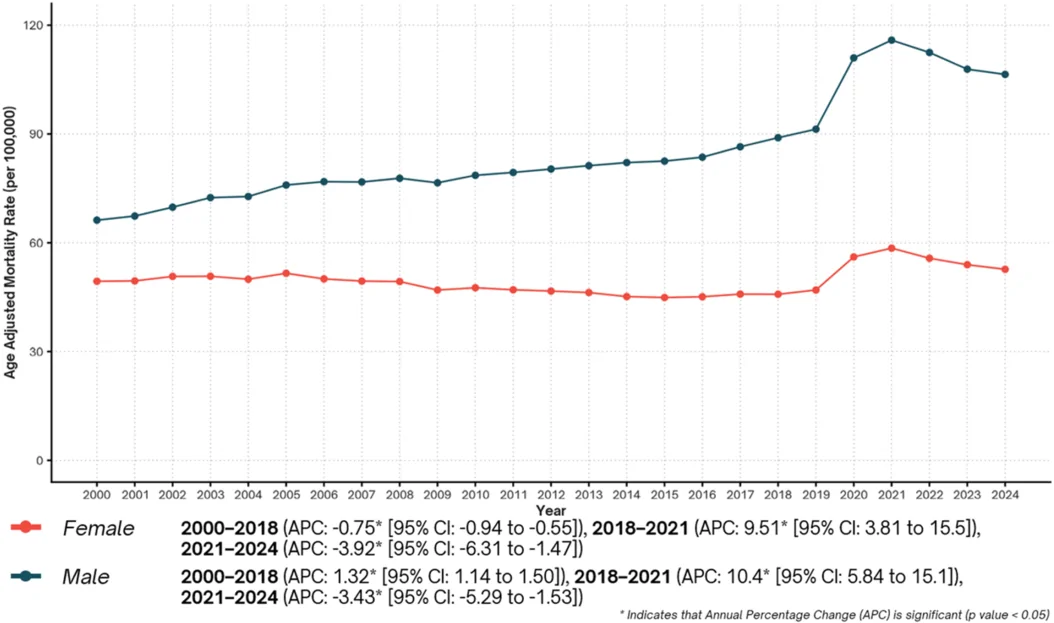
Coronary artery disease and hypertension‐related age‐adjusted mortality rates among US adults per 100 000 stratified by sex, 2000 to 2024.

Men represented 55.63% of CAD and hypertension‐related deaths, while women accounted for 44.37% from 2000 to 2024. Females had consistently lower AAMR compared to males throughout the study period. The total number of deaths in females was 1 675 576, compared to 2 100 820 in males. Male mortality showed a significant and gradual increase from 2000 to 2018 (APC: 1.32; 95% CI: 1.14 to 1.50; *p* < 0.000001), followed by a steep and significant increase from 2018 to 2021 (APC: 10.35; 95% CI: 5.84 to 15.06; *p* = 0.000115), and then a significant decline from 2021 to 2024 (APC: −3.43; 95% CI: −5.29 to −1.53; *p* = 0.001502). In 2000, female and male AAMR were 49.39 (95% CI: 48.98 to 49.81) and 66.23 (95% CI: 65.63 to 66.84), respectively. By 2024, male AAMR was approximately twice that of females (106.43 vs. 52.68). Female AAMR showed a significant gradual decline from 2000 to 2018 (APC: −0.75; 95% CI: −0.94 to −0.55; *p* < 0.000001), followed by a steep and significant increase from 2018 to 2021 (APC: 9.51; 95% CI: 3.81 to 15.51; *p* = 0.002267), and then a significant decline from 2021 to 2024 (APC: −3.92; 95% CI: −6.31 to −1.47; *p* = 0.003830) (Figure 2) (Supporting Information S1: Table 2).

### 3.3 Mortality Trends Stratified by Race

When stratified by race, the highest average AAMR was observed among NH Black or African American adults (96.90; 95% CI: 96.62–97.17) with 509 581 deaths, followed by American Indian or Alaska Native adults (68.40; 95% CI: 67.42–69.37), NH White adults (63.69; 95% CI: 63.61–63.76), Hispanic or Latino adults (57.40; 95% CI: 57.17–57.64), and Asian or Pacific Islander adults (42.68; 95% CI: 42.40–42.96).

**Figure 3:**
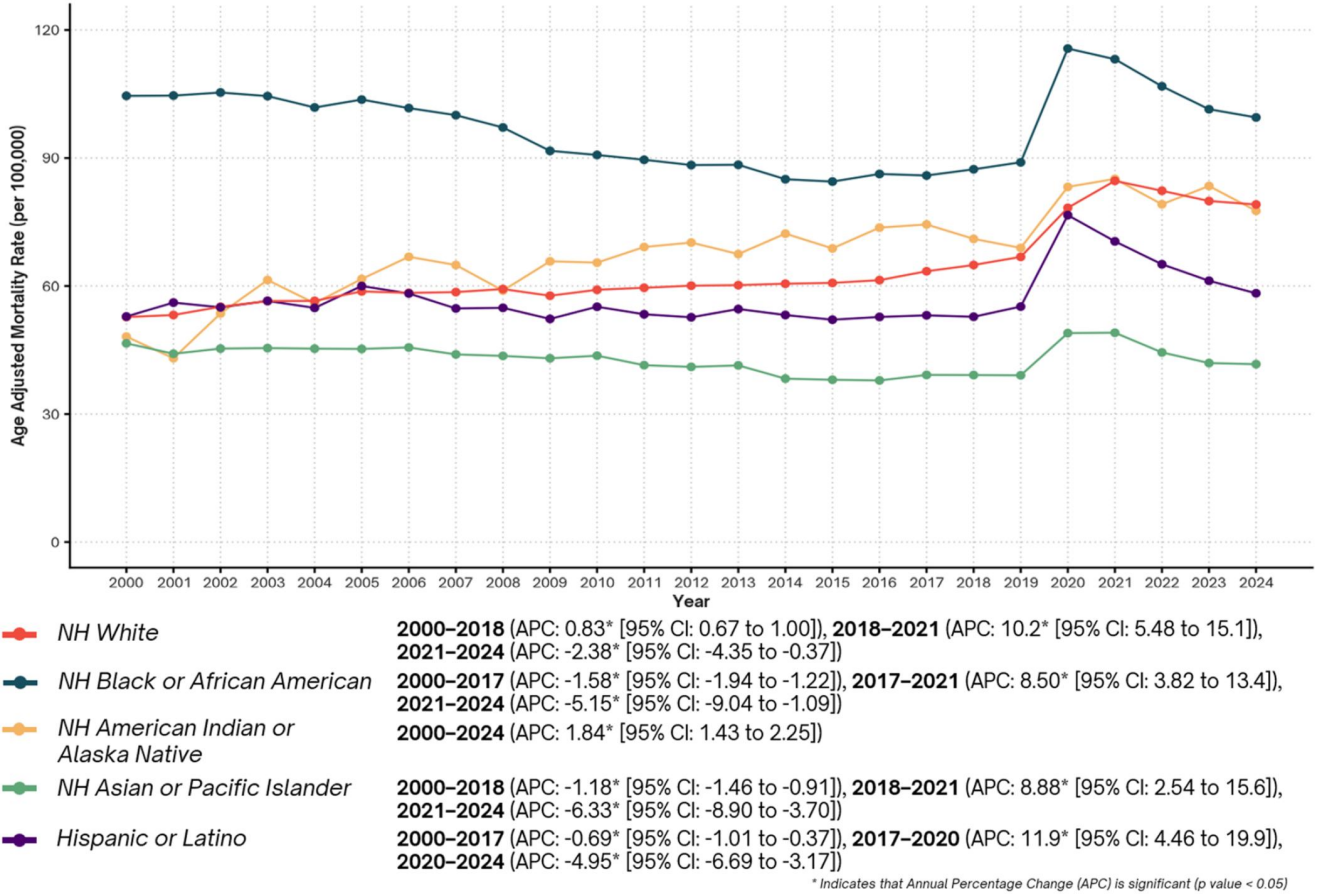
Coronary artery disease and hypertension‐related age‐adjusted mortality rates among US adults per 100 000 stratified by Race, 2000 to 2024.

Significant increases in AAMR were observed among American Indian or Alaska Native adults throughout the study period (APC: 1.84; 95% CI: 1.43–2.25; *p* < 0.000001). Among NH White adults, AAMR increased gradually from 2000 to 2018 (APC: 0.83; 95% CI: 0.67–1.00; *p* < 0.000001), followed by a steeper increase from 2018 to 2021 (APC: 10.16; 95% CI: 5.48–15.05; *p* = 0.000205), and then declined from 2021 to 2024 (APC: −2.38; 95% CI: −4.35 to −0.37; *p* = 0.023375). Among NH African American adults, AAMR declined from 2000 to 2017 (APC: −1.58; 95% CI: −1.94 to −1.22; *p* < 0.000001), increased from 2017 to 2021 (APC: 8.50; 95% CI: 3.82–13.39; *p* = 0.001145), and subsequently declined through 2024 (APC: −5.15; 95% CI: −9.04 to −1.09; *p* = 0.016499). Asian or Pacific Islander adults showed a significant decline from 2000 to 2018 (APC: −1.18; 95% CI: −1.46 to −0.91; *p* < 0.000001), followed by a significant increase through 2021 (APC: 8.88; 95% CI: 2.54–15.62; *p* = 0.008239) and a subsequent decline through 2024 (APC: −6.33; 95% CI: −8.90 to −3.70; *p* = 0.000116). Hispanic or Latino adults similarly experienced a significant decline from 2000 to 2017 (APC: −0.69; 95% CI: −1.01 to −0.37; *p* = 0.000309), followed by a significant increase from 2017 to 2020 (APC: 11.93; 95% CI: 4.46–19.93; *p* = 0.003102) and a significant decline from 2020 to 2024 (APC: −4.95; 95% CI: −6.69 to −3.17; *p* = 0.000022) (Figure 3) (Supporting Information S1: Tables 2).

### 3.4 Mortality Trends Stratified by Urbanization

**Figure 4:**
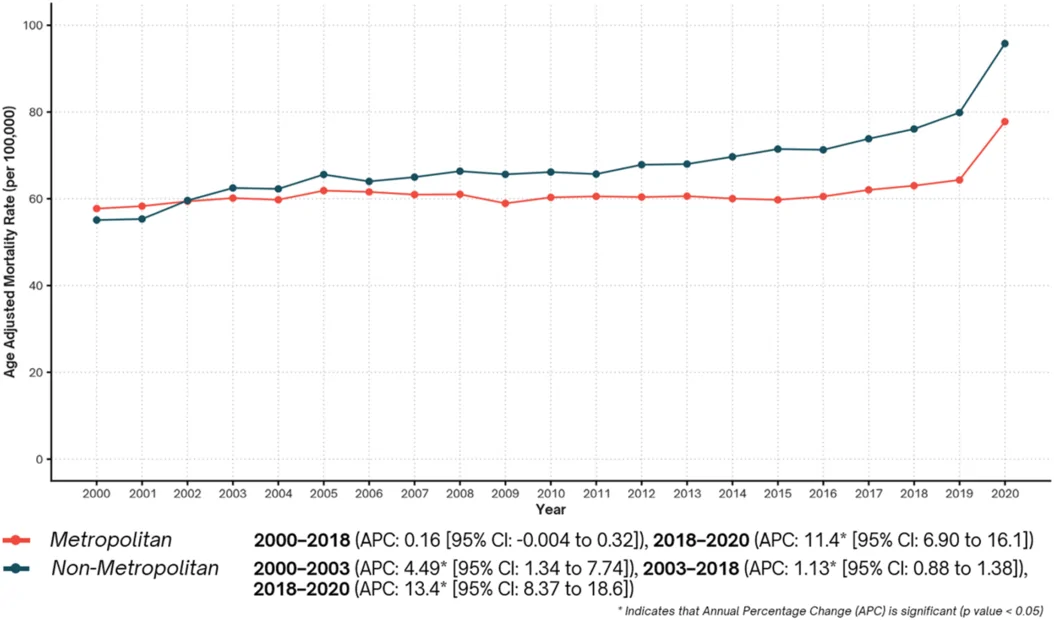
Coronary artery disease and hypertension‐related age‐adjusted mortality rates among US adults per 100 000 stratified by Urbanization, 2000 to 2024.

When stratified by urbanization, non‐metropolitan (rural) areas had a higher average burden of CAD and hypertension‐related mortality among adults than metropolitan (urban) areas over the study period, with average AAMRs of 68.15 and 61.55 per 100 000, respectively. In non‐metropolitan areas, AAMR increased significantly throughout the study period, with an AAPC of 2.79% (95% CI: 2.17–3.42; *p* < 0.000001). This increase was characterized by significant rises from 2000 to 2003 (APC: 4.49%; 95% CI: 1.34–7.74; *p* = 0.008418), 2003 to 2018 (APC: 1.13%; 95% CI: 0.88–1.38; *p* < 0.000001), and a steeper increase from 2018 to 2020 (APC: 13.36%; 95% CI: 8.37–18.59; *p* = 0.000044). In metropolitan areas, the overall AAMR also increased significantly, with an AAPC of 1.23% (95% CI: 0.82–1.64; *p* < 0.000001), although the trend from 2000 to 2018 was not statistically significant (APC: 0.16%; 95% CI: −0.00–0.32; *p* = 0.054559), followed by a significant and steeper increase from 2018 to 2020 (APC: 11.42%; 95% CI: 6.90–16.14; *p* = 0.000045) (Figure 4) (Supporting Information S1: Table 2).

### 3.5 Mortality Trends Stratified by Census Region and States

**Figure 5:**
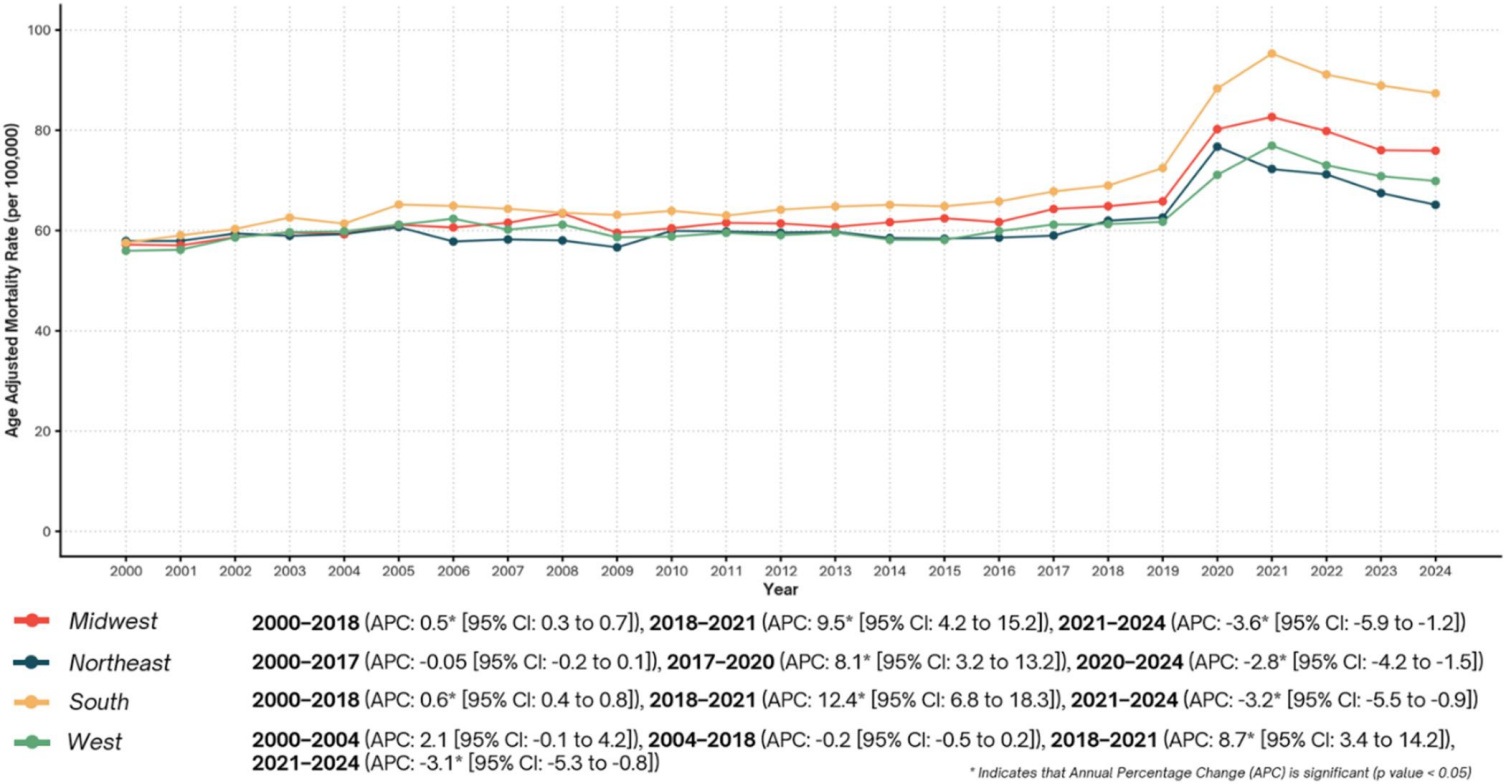
Coronary artery disease and hypertension‐related age‐adjusted mortality rates among US adults per 100 000 stratified by census region, 2000 to 2024.

All four census regions demonstrated an overall increasing trend in CAD and hypertension‐related mortality in adults from 2000 to 2024. The South had the highest average AAMR (70.25 per 100 000) and showed the greatest significant increase (AAPC: 1.53%; 95% CI: 0.85–2.22; *p* = 0.000010). The Midwest also demonstrated a significant increase (AAPC: 1.04%; 95% CI: 0.38–1.71; *p* = 0.002053), followed by the West (AAPC: 0.91%; 95% CI: 0.18–1.64; *p* = 0.014185). In contrast, the Northeast had the lowest average AAMR (61.57 per 100 000) and demonstrated a non‐significant overall increase (AAPC: 0.46%; 95% CI: −0.13–1.06; *p* = 0.127527). Overall, the South and Midwest experienced the steepest significant increases in CAD mortality (Figure 5) (Supporting Information S2: Figure 1; Supporting Information S1: Table 2).

**Figure 6:**
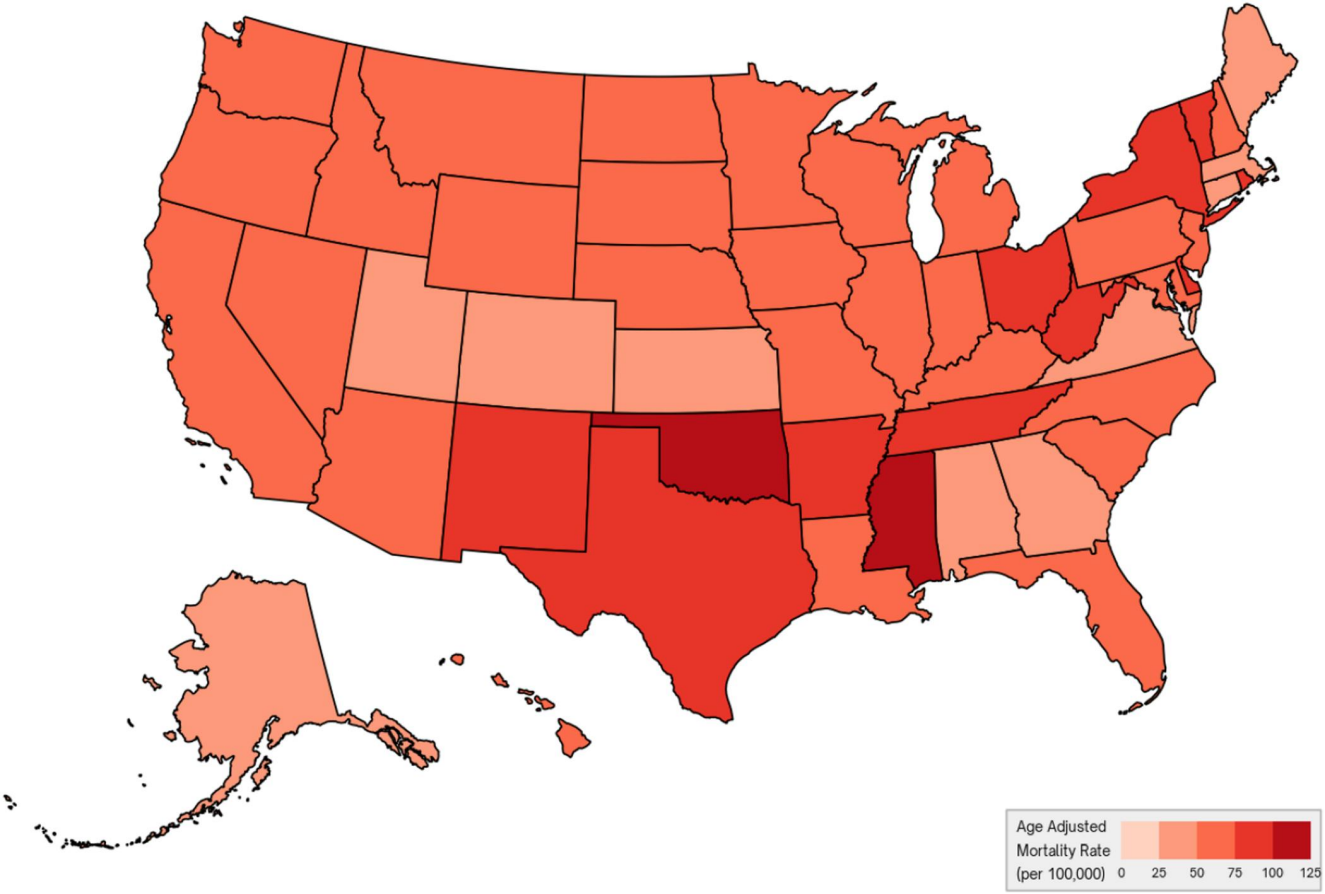
Coronary artery disease and hypertension‐related age‐adjusted mortality rates among US adults per 100 000 stratified by States, 2000 to 2024.

State‐level CAD and hypertension‐related mortality among adults varied substantially across the United States. States in the highest 10th percentile for CAD‐related mortality included the District of Columbia (AAMR: 121.88), Mississippi (107.76), Oklahoma (102.62), Vermont (98.97), and West Virginia (91.69). In contrast, states in the lowest 10th percentile included Utah (35.46), Massachusetts (40.44), Connecticut (44.44), Alabama (46.25), and Maine (46.84) (Figure 6) (Supporting Information S1: Table 2).

### 3.6 Mortality Trends Stratified by Age

Among adults aged 25–44 years, mortality increased significantly throughout the early and middle study periods. From 2000 to 2006, the AAMR increased by 4.93% annually (95% CI: 3.31–6.57; *p* = 0.000011), followed by a significant increase of 2.58% annually from 2006 to 2018 (95% CI: 2.02–3.15; *p* < 0.000001). The rate increased more sharply from 2018 to 2021, with an APC of 8.72% (95% CI: 1.59–16.36; *p* = 0.019321). From 2021 to 2024, mortality showed a nonsignificant decline (APC: −1.05%; 95% CI: −3.98–1.96; *p* = 0.460631). Overall, the AAPC for 2000–2024 was 3.45% (95% CI: 2.47–4.44; *p* < 0.000001).

Among adults aged 45–64 years, mortality increased significantly by 1.99% annually from 2000 to 2018 (95% CI: 1.84–2.14; *p* < 0.000001). This increase accelerated substantially during 2018–2021, with an APC of 8.87% (95% CI: 4.94–12.95; *p* = 0.000141). From 2021 to 2024, the AAMR declined significantly by 3.71% annually (95% CI: −5.40 to −1.99; *p* = 0.000314). The overall AAPC from 2000 to 2024 was 2.09% (95% CI: 1.60–2.58; *p* < 0.000001).

Among adults aged 65–85+ years, the mortality trend remained essentially stable from 2000 to 2018 (APC: 0.0009%; 95% CI: −0.19–0.19; *p* = 0.991960). This was followed by a marked and significant increase from 2018 to 2021, with an APC of 10.47% (95% CI: 5.23–15.97; *p* = 0.000458). From 2021 to 2024, mortality declined significantly by 3.57% annually (95% CI: −5.72 to −1.36; *p* = 0.003517). Despite these fluctuations, the overall AAPC for 2000–2024 was 0.79% (95% CI: 0.16–1.44; *p* = 0.014749).

Overall, all three age groups demonstrated significant increases in mortality over the full 2000–2024 period, as reflected by their positive AAPCs. The most pronounced acceleration occurred during 2018–2021 across all age groups, with the largest increase among adults aged 65–85+ years (APC: 10.47%), followed by those aged 45–64 years (8.87%) and 25–44 years (8.72%). Mortality subsequently declined significantly among the two older age groups from 2021 to 2024, whereas the decline among adults aged 25–44 years was not statistically significant (Supporting Information S2: Figure 2; Supporting Information S1: Table 2).

### 3.7 Mortality Trends Stratified by Place of Death

Analysis of place of death among adults with CAD and hypertension revealed that the majority of deaths occurred at the decedent's home or in healthcare facilities. Deaths at the decedent's home accounted for the largest proportion (36.00%), followed by inpatient medical facility deaths (22.11%) and deaths in nursing homes or long‐term care facilities (18.79%). Outpatient or emergency department deaths accounted for 13.54% of deaths, while hospice facilities accounted for 3.11%. Deaths occurring in other locations represented 5.19%, and 1.05% of individuals were dead on arrival. Deaths with an unknown place of death (0.17%) or unknown status within a medical facility (0.04%) were minimal (Supporting Information S2: Figure 3).

### 3.8 Subgroup Analysis by CAD Subtypes

Subgroup analysis of AAMRs by CAD subtype from 2000 to 2024 demonstrated distinct temporal patterns.

Angina pectoris showed a significant decline from 2000 to 2011 (APC, −10.35%; 95% CI, −11.76 to −8.91; *p* < 0.000001), followed by a significant increase from 2011 to 2021 (APC, 9.69%; 95% CI, 7.52 to 11.91; *p* < 0.000001). From 2021 to 2024, AAMR declined modestly, but this change was not statistically significant (APC, −2.92%; 95% CI, −9.60 to 4.25; *p* = 0.392666). Overall, angina pectoris mortality showed a significant decrease across the study period (AAPC, −1.51%; 95% CI, −2.80 to −0.20; *p* = 0.023653).

Acute MI and subsequent MI demonstrated a significant decline from 2000 to 2012 (APC, −2.09%; 95% CI, −2.41 to −1.77; *p* < 0.000001), followed by a statistically non‐significant plateau from 2012 to 2018 (APC, −0.37%; 95% CI, −1.51 to 0.80; *p* = 0.509379). This was followed by a significant increase from 2018 to 2021 (APC, 8.12%; 95% CI, 3.08 to 13.40; *p* = 0.003458), and subsequently a significant decline from 2021 to 2024 (APC, −7.50%; 95% CI, −9.56 to −5.39; *p* < 0.000003). Overall, mortality declined significantly during 2000–2024 (AAPC, −1.14%; 95% CI, −1.80 to −0.47; *p* = 0.000874).

CIHD remained the predominant contributor to CAD mortality. Its AAMR increased significantly from 2000 to 2018 (APC, 0.66%; 95% CI, 0.48 to 0.85; *p* < 0.000001), followed by a pronounced and significant increase from 2018 to 2021 (APC, 10.18%; 95% CI, 5.18 to 15.41; *p* < 0.000387). A significant decline subsequently occurred from 2021 to 2024 (APC, −2.76%; 95% CI, −4.87 to −0.61; *p* = 0.015193). Despite the recent decline, CIHD demonstrated a significant overall increase across the study period (AAPC, 1.37%; 95% CI, 0.75 to 1.99; *p* < 0.000013).

Other acute ischemic heart diseases remained the least prominent subtype, with an average AAMR of 1.03 per 100 000. Although several periods showed increases or decreases, none of the segment‐specific APCs reached statistical significance. The overall trend was also not statistically significant (AAPC, 2.75%; 95% CI, −0.38 to 5.99; *p* = 0.085863) (Supporting Information S2: Figure 4; Supporting Information S1: Table 2).

### 3.9 Subgroup Analysis by Hypertension Subtypes

Subgroup analysis revealed heterogeneous temporal trends across different hypertensive disease subtypes.

Essential Hypertension: The AAMR decreased from 47.86 per 100 000 in 2000 to 45.99 per 100 000 in 2024, with an overall non‐significant AAPC of −0.30% (*p* = 0.642592). The trend initially increased non‐significantly from 2000 to 2008 (APC: 0.86%), followed by a significant decline from 2008 to 2018 (APC: −2.08%; *p* = 0.000180). A non‐significant increase occurred from 2018 to 2021 (APC: 9.01%), followed by a significant decline from 2021 to 2024 (APC: −6.13%; *p* = 0.009564).

Hypertensive Heart Disease: The AAMR increased substantially from 7.51 per 100 000 in 2000 to 21.27 per 100 000 in 2024, with a significant overall AAPC of 4.35% (*p* < 0.000001). Following a non‐significant decline from 2000 to 2002 (APC: –2.57%), mortality increased significantly from 2002 to 2012 (APC: 3.21%; *p* < 0.000001), 2012–2018 (APC: 6.25%; *p* < 0.000001), and 2018–2021 (APC: 12.44%; *p* = 0.000009). From 2021 to 2024, the increase was no longer statistically significant (APC: 1.46%; *p* = 0.050368).

Hypertensive Renal Disease: The AAMR increased from 1.73 per 100 000 in 2000 to 8.62 per 100 000 in 2024, with a significant overall AAPC of 8.83% (*p* < 0.000001), indicating a sustained increasing trend throughout the study period.

Hypertensive Heart and Renal Disease: The AAMR increased from 0.57 per 100 000 in 2000 to 2.38 per 100 000 in 2024, with a significant overall AAPC of 6.57% (*p* < 0.000001). Mortality initially declined significantly from 2000 to 2012 (APC: −2.86%; *p* = 0.007280), followed by a marked and significant increase from 2012 to 2024 (APC: 16.92%; *p* < 0.000001).

Overall, mortality trends varied substantially across hypertensive disease subtypes, with both declining and increasing patterns observed across different time periods. Essential hypertension showed a non‐significant overall decline in mortality, whereas hypertensive heart disease, hypertensive renal disease, and hypertensive heart and renal disease demonstrated significant overall increases over the study period. Hypertensive renal disease had the highest overall increase (AAPC: 8.83%), while hypertensive heart and renal disease exhibited the steepest segment‐specific increase (APC: 16.92% from 2012 to 2024) (Supporting Information S2: Figure 5; Supporting Information S1: Table 2).

## 4 Discussion

This nationwide analysis examined mortality trends due to CAD and hypertension among adults aged ≥ 25 years in the United States between 2000 and 2024 and identified several important findings. First, mortality rates for deaths due to both CAD and hypertension increased significantly over the study period, with the AAMR rising more than eightfold. Second, notable disparities were observed across sex, age groups, racial and ethnic populations, geographic regions, and rural‐urban settings. Third, subtype analyses demonstrated that CIHD and AMI accounted for most of the CAD mortality, while hypertensive heart and renal disease showed rapidly increasing trends, suggesting an evolving burden of hypertensive cardiovascular complications. These findings should be interpreted as population‐level mortality patterns derived from death certificate coding rather than evidence of causal relationships between hypertension and CAD mortality (Central Illustration 1).

**Central Illustration 1:**
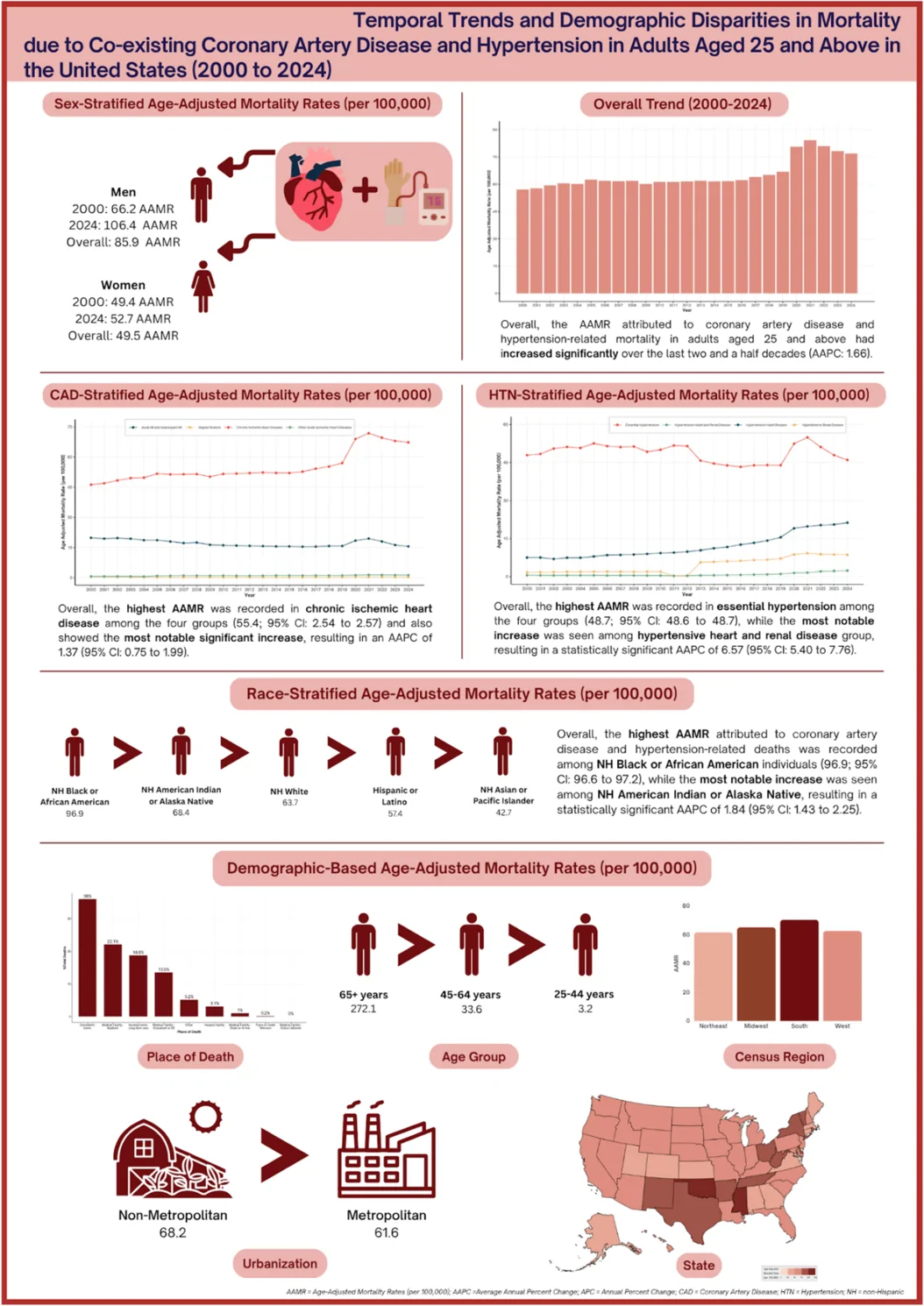
Central illustration demonstrating various temporal trends in coronary artery disease and hypertension‐related age‐adjusted mortality rates (per 100 000) among adults aged 25 and above in the United States, 2000 to 2024.

The rising mortality burden observed in this study highlights the persistent coexistence of CAD and hypertension in cardiovascular deaths across the United States. Hypertension remains one of the most significant modifiable risk factors for atherosclerotic cardiovascular disease and contributes to endothelial dysfunction, vascular remodeling, and accelerated atherosclerosis [17, 18]. Despite improvements in cardiovascular prevention and treatment over recent decades, hypertension prevalence remains high in the United States, and blood pressure control rates have stagnated or declined in certain populations [19, 20]. These trends, combined with increasing rates of obesity, diabetes, and metabolic syndrome, may have contributed to the observed increase in CAD and hypertension‐related mortality [21].

Sex‐based differences were prominent in this analysis, with males accounting for many deaths and demonstrating significantly higher mortality rates compared with females. Previous studies have consistently shown that men experience earlier onset and higher incidence of CAD, potentially due to differences in cardiovascular risk profiles, hormonal influences, and lifestyle factors [22]. Estrogen is believed to exert protective cardiovascular effects in premenopausal women, which may partially explain lower CAD mortality among females [23]. Additionally, differences in healthcare‐seeking behavior and risk factor management may further contribute to sex‐based disparities in cardiovascular outcomes [24]. Nevertheless, these explanations remain inferential because the present study design does not directly evaluate biological or behavioral mechanisms. Age‐stratified analyses demonstrated that older adults aged 65 years and above carried the greatest mortality burden, consistent with the well‐established relationship between aging and CAD risk [25]. However, the significant rise in mortality among younger adults aged 25–44 years is particularly concerning. Recent epidemiological studies have reported increasing prevalence of hypertension and cardiometabolic risk factors among younger populations, including obesity, diabetes, and sedentary lifestyles [26]. Early exposure to these risk factors may accelerate atherosclerotic progression and increase lifetime cardiovascular risk [27]. While these factors may partially explain the observed trends, the current analysis cannot determine the individual‐level drivers underlying increasing mortality rates in younger adults.

Substantial racial and ethnic disparities were observed in CAD and hypertension mortality among adults. NH Black individuals exhibited the highest mortality rates, consistent with prior research demonstrating disproportionate hypertension prevalence and poorer blood pressure control in this population [28, 29]. Structural determinants of health, including socioeconomic inequalities, differences in healthcare access, and disparities in preventive care, likely contribute to these persistent inequities in cardiovascular outcomes [30]. NH American Indian and Alaska Native populations also demonstrated significant increases in mortality over time, which may reflect rising cardiometabolic disease burden and limited access to healthcare resources in certain communities [31].

Regional analyses revealed that the Southern United States experienced the highest mortality rates and the steepest increases over time. This finding aligns with previous literature describing the “stroke belt,” a region characterized by disproportionately high cardiovascular morbidity and mortality [32, 33]. Higher prevalence of hypertension, diabetes, obesity, and smoking in the Southern states likely contributes to these patterns [34]. Additionally, socioeconomic disparities and differences in healthcare access may further exacerbate regional variations in cardiovascular outcomes. However, these factors were not directly measured in the present study and therefore should be interpreted as possible explanations rather than confirmed causes.

Urban‐rural disparities were also evident, with rural populations demonstrating consistently higher CAD mortality compared with urban populations. Prior studies have documented widening rural‐urban gaps in cardiovascular mortality across the United States [35]. Factors contributing to these disparities may include limited access to specialty care, shortages of healthcare providers, delayed diagnosis of cardiovascular disease, and reduced availability of preventive healthcare services in rural areas [36]. Because urbanization data in CDC WONDER were only available through 2020, interpretation of these trends should be made within that temporal limitation. State‐level variation further highlights the heterogeneity of mortality patterns across the United States. States such as Mississippi and West Virginia have historically reported some of the highest cardiovascular mortality rates in the nation, reflecting a higher prevalence of cardiovascular risk factors and socioeconomic disadvantages [37]. In contrast, states with stronger preventive healthcare systems and better cardiovascular risk factor control, such as Massachusetts and Utah, tend to demonstrate lower mortality rates [38]. These geographic differences likely represent complex interactions among demographic, socioeconomic, healthcare access, and behavioral factors that could not be directly evaluated in this study.

Analysis of place of death indicated that most deaths occurred at home or in healthcare facilities, suggesting that CAD in hypertensive individuals frequently progresses as a chronic condition culminating outside acute hospital settings. Similar patterns have been observed in other cardiovascular mortality studies, reflecting both the chronic nature of cardiovascular disease and increasing utilization of home and hospice‐based end‐of‐life care [39].

Subtype analyses revealed that CIHD and AMI accounted for the majority of CAD‐related deaths. A marked increase in mortality rates was observed between 2018 and 2021. Although this temporal pattern overlaps with the COVID‐19 pandemic period and may reflect healthcare disruptions, delayed presentations, or indirect cardiovascular effects associated with the pandemic, the present study was not designed to directly evaluate these mechanisms, and causal attribution cannot be established [40]. Furthermore, the steady increase in hypertensive heart disease and hypertensive renal disease highlights the progressive cardiovascular and renal complications associated with long‐standing hypertension [41].

## 5 Limitations

Several limitations should be considered. First, CDC WONDER relies on death certificate data, which may be subject to misclassification or inaccuracies in reporting underlying causes of death. CAD and hypertension diagnoses were identified from death certificate coding rather than clinically adjudicated medical records, and therefore the findings reflect documented mortality associations rather than confirmed disease causation at the individual level. Second, the ecological and retrospective nature of this analysis precludes assessment of individual‐level associations and does not permit causal inference regarding the relationship between hypertension and CAD mortality. Third, the database lacks detailed clinical variables, including hypertension severity, duration of disease, medication adherence, blood pressure control, socioeconomic status, comorbidities, and access to healthcare, limiting the ability to evaluate mechanisms underlying observed disparities. Fourth, race and ethnicity data recorded on death certificates may be subject to misclassification. Additionally, CDC WONDER combined Asian and Pacific Islander populations after 2020, which may reduce the precision of subgroup analyses and obscure differences between these populations. Fifth, some subgroup estimates may be unstable because of small cell counts or suppression of rare events, particularly in less populous racial or geographic subgroups. Changes in death certification practices, ICD‐10 coding patterns, and reporting practices over time may also have influenced the observed temporal trends. Finally, mortality increases observed between 2018 and 2021 coincided with the COVID‐19 pandemic. Because COVID‐19‐related deaths and indirect pandemic‐related healthcare disruptions could not be separately excluded within the database, some observed mortality trends may have been influenced by pandemic‐related factors.

## 6 Conclusion

In conclusion, this nationwide analysis demonstrated increasing mortality rates involving CAD and hypertension among US adults over the past two decades, with substantial demographic and geographic disparities across sex, race and ethnicity, age groups, regions, and rural‐urban populations. Because these findings are derived from death certificate data, they should be interpreted as population‐level mortality associations rather than evidence of direct causality between hypertension and CAD mortality. Nevertheless, the findings underscore the ongoing public health burden associated with coexisting hypertension and CAD and highlight the need for improved cardiovascular risk reduction, equitable healthcare access, and further longitudinal research to better understand the drivers of these mortality patterns.

## Author Contributions

**Muhammad Shayan Tariq:** contributed to conceptualization, writing − original draft, writing – review and editing, visualization, formal analysis, and project administration. **Rai Muhammad Umar Khan:** contributed to investigation, software, writing – original draft, data curation, and resources. **Asad Ullah Farooq:** contributed to writing – original draft, writing − review and editing, and formal analysis. **Kinza Raza:** contributed to writing − original draft, writing – review and editing, and resources. **Maryam Athar:** contributed to validation and methodology. **Shumail Tariq** and **Muhammad Raza Asif:** contributed to visualization and data curation. **Noor Ul Huda Ramzan:** contributed to writing – review and editing and data curation. **Ameer Haider Cheema:** contributed to investigation, software, and resources.

## Funding

The authors have nothing to report.

## Ethics Statement

Not applicable because of the use of a de‐identified, anonymized, public database.

## Consent

The authors have nothing to report.

## Conflicts of Interest

The authors declare no conflicts of interest.

## Supporting information


Supporting File 1

Supporting File 2

## Data Availability

The data supporting the findings of this study are publicly available from the CDC WONDER database.
