# Supplementary material for: Temporal Trends and Demographic Disparities in Mortality Due to Coronary Artery Disease and Hypertension in Adults Aged 25 and Above in the United States (2000 to 2024)
Source: Clin Cardiol. 2026 Sep 4;49(9):e70465. doi: 10.1002/clc.70465 (PMC13543300; doi:10.1002/clc.70465)
Supplement: Supplementary file 1 [file CLC-49-e70465-s001.docx]

**SUPPLEMENTAL TABLES/FIGURES LEGENDS**

**Supplemental Table 1.** Absolute number and percent total deaths of coronary artery disease and hypertension-related deaths among aged 25 and above stratified by overall, gender, race/ethnicity, age group, place of death, urbanization, and regions in the United States, 2000 to 2024.

**Supplemental Table 2.** Annual percent change (APC) of coronary artery disease and hypertension-related age-adjusted mortality rates (per 100,000) among adults aged 25 and above in the United States, 2000 to 2024.

**Supplemental Table 3.** Overall and sex-stratified coronary artery disease and hypertension-related age-adjusted mortality rates (per 100,000) among adults aged 25 and above in the United States, 2000 to 2024.

**Supplemental Table 4.** Race/Ethnicity-stratified coronary artery disease and hypertension-related age-adjusted mortality rates (per 100,000) among adults aged 25 and above in the United States, 2000 to 2024.

**Supplemental Table 5.** Urbanization-stratified coronary artery disease and hypertension-related age-adjusted mortality rates (per 100,000) among adults aged 25 and above in the United States, 2000 to 2020.

**Supplemental Table 6.** Census Region-stratified coronary artery disease and hypertension-related age-adjusted mortality rates (per 100,000) among adults aged 25 and above in the United States, 2000 to 2024.

**Supplemental Table 7.** State-stratified coronary artery disease and hypertension-related age-adjusted mortality rates (per 100,000) among adults aged 25 and above in the United States, 2000 to 2024.

**Supplemental Table 8.** Age group-stratified coronary artery disease and hypertension-related crude mortality rates (per 100,000) among adults aged 25 and above in the United States, 2000 to 2024.

**Supplemental Table 9.** Coronary artery disease subgroups-stratified coronary artery disease and hypertension-related age-adjusted mortality rates (per 100,000) among adults aged 25 and above in the United States, 2000 to 2024.

**Supplemental Table 10.** Hypertension subgroups-stratified coronary artery disease and hypertension-related age-adjusted mortality rates (per 100,000) among adults aged 25 and above in the United States, 2000 to 2024.

**Supplementary Figure 1.** Census region-stratified coronary artery disease and hypertension-related age-adjusted mortality rates (per 100,000) among adults aged 25 and above in the United States, 2000 to 2024.

**Supplementary Figure 2.** Coronary artery disease and hypertension-related age-adjusted mortality rates stratified by age groups 25 to 85+ years in the United States, 2000 to 2024.

**Supplementary Figure 3.** Percent total deaths of coronary artery disease and hypertension-related mortality stratified by place of death among adults aged 25 and above in the United States, 2000 to 2024.

**Supplementary Figure 4.** Trends in coronary artery disease and hypertension-related age-adjusted mortality rates (per 100,000) stratified by coronary artery disease subgroups among adults aged 25 and above in the United States, 2000 to 2024.

**Supplementary Figure 5.** Trends in coronary artery disease and hypertension-related age-adjusted mortality rates (per 100,000) stratified by hypertension subgroups among adults aged 25 and above in the United States, 2000 to 2024.

| **Variables** | **Deaths** | **% of Total Deaths** |
| --- | --- | --- |
| Overall | 3776396 | 100% |
| **Gender** | | |
| Female | 1675576 | 44% |
| Male | 2100820 | 56% |
| **Race** | | |
| NH White | 2875561 | 76.2% |
| NH Black or African American | 509581 | 13.5% |
| NH American Indian or Alaska Native | 21756 | 0.6% |
| NH Asian or Pacific Islander | 96444 | 2.6% |
| Hispanic or Latino | 255920 | 6.8% |
| **Age Groups** | | |
| 25-44 years | 63737 | 1.7% |
| 45-64 years | 709537 | 18.8% |
| 65+ years | 3003122 | 79.5% |
| **Place of Deaths** | | |
| Medical Facility - Inpatient | 834923 | 22% |
| Medical Facility - Outpatient or ER | 511492 | 13.5% |
| Medical Facility - Dead on Arrival | 39614 | 1.1% |
| Medical Facility - Status Unknown | 1326 | 0.04% |
| Decedent's home | 1359658 | 36% |
| Nursing home/long term care | 117514 | 3.1% |
| Hospice facility | 709637 | 19% |
| Other | 195900 | 5.2% |
| Place of Death Unknown | 6332 | 0.2% |
| **Urbanization** | | |
| Metropolitan Areas | 2350118 | 81% |
| Non-Metropolitan Areas | 551654 | 19% |
| **Regions** | | |
| Northeast | 693193 | 18% |
| Midwest | 833907 | 22% |
| South | 1475176 | 39% |
| West | 774120 | 21% |
| **CAD Subgroups** | | |
| Angina Pectoris | 15492 | 0.4% |
| Acute MI and Subsequent MI | 977460 | 23% |
| Other Acute Ischemic Heart Diseases | 59930 | 1.4% |
| Chronic Ischemic Heart Disease | 3185028 | 75.2% |
| **HTN Subgroups** | | |
| Essential Hypertension | 2775377 | 73% |
| Hypertensive Heart Disease | 714662 | 19% |
| Hypertensive Renal Disease | 271586 | 7% |
| Hypertensive Heart and Renal Disease | 51146 | 1% |
| *CAD = Coronary Artery Disease; HTN = Hypertension; NH: Non-Hispanic* | | |

**Supplemental Table 1.** Absolute number and percent total deaths of coronary artery disease and hypertension-related deaths among aged 25 and above stratified by overall, gender, race/ethnicity, age group, place of death, urbanization, and regions in the United States, 2000 to 2024.

| **Year Interval** | **APC (95% CI)** |
| --- | --- |
| **Overall** | |
| 2000-2018 | 0.4* (0.2 to 0.6) |
| 2018–2021 | 10.1* (5.5 to 15.0) |
| 2021–2024 | -3.5* (-5.4 to -1.6) |
| **Gender** | |
| **Male** | |
| 2000–2018 | 1.3* (1.1 to 1.5) |
| 2018–2021 | 10.4* (5.8 to 15.1) |
| 2021–2024 | -3.4* (-5.3 to -1.5) |
| **Female** | |
| 2000-2018 | -0.8* (-0.9 to -0.6) |
| 2018–2021 | 9.5* (3.8 to 15.5) |
| 2021–2024 | -3.9* (-6.3 to -1.5) |
| **Race** | |
| **NH American Indian or Alaska Native** | |
| 2000-2024 | 1.8* (1.4 to 2.3) |
| **NH Asian or Pacific Islander** | |
| 2000-2018 | -1.2* (-1.5 to -0.9) |
| 2018–2021 | 8.9* (2.5 to 15.6) |
| 2021–2024 | -6.3* (-8.9 to -3.7) |
| **NH Black or African American** | |
| 2000-2017 | -1.6* (-1.9 to -1.2) |
| 2017–2021 | 8.5* (3.8 to 13.4) |
| 2021–2024 | -5.2* (-9.0 to -1.1) |
| **NH White** | |
| 2000-2018 | 0.8* (0.7 to 1.0) |
| 2018–2021 | 10.2* (5.5 to 15.1) |
| 2021–2024 | 2.38* (-4.4 to -0.4) |
| **Hispanic or Latino** | |
| 2000-2017 | -0.7* (-1.0 to -0.4) |
| 2017–2021 | 11.9* (4.5 to 19.9) |
| 2021–2024 | -5.0* (-6.7 to -3.2) |
| **Age Groups** | |
| **Young-Aged (25-44 years)** | |
| 2000-2006 | 4.9* (3.3 to 6.6) |
| 2006-2018 | 2.6* (2.0 to 3.2) |
| 2018–2021 | 8.7* (1.6 to 16.4) |
| 2021–2024 | -1.1 (-4.0 to 2.0) |
| **Middle-Aged (45-64 years)** | |
| 2000-2018 | 2.0* (1.8 to 2.1) |
| 2018–2021 | 8.9* (4.9 to 13.0) |
| 2021–2024 | -3.7* (-5.4 to -2.0) |
| **Advanced-Aged (65+ years)** | |
| 2000-2018 | 0.001 (-0.2 to 0.2) |
| 2018–2021 | 10.5* (5.2 to 16.0) |
| 2021–2024 | -3.6* (-5.7 to -1.4) |
| **Urbanization** | |
| **Metropolitan** | |
| 2000-2018 | 0.2 (-0.004 to 0.3) |
| 2018–2024 | 11.4* (6.9 to 16.1) |
| **Non-Metropolitan** | |
| 2000-2003 | 4.5* (1.3 to 7.7) |
| 2003-2018 | 1.1* (0.9 to 1.4) |
| 2018–2024 | 13.4* (8.4 to 18.6) |
| **Regions** | |
| **Northeast** | |
| 2000-2017 | -0.05 (-0.2 to 0.1) |
| 2017–2020 | 8.1* (3.2 to 13.2) |
| 2020–2024 | -2.8* (-4.2 to -1.5) |
| **Midwest** | |
| 2000-2018 | 0.5* (0.3 to 0.7) |
| 2018–2021 | 9.5* (4.2 to 15.2) |
| 2021–2024 | -3.6* (-5.9 to -1.2) |
| **South** | |
| 2000-2018 | 0.6* (0.4 to 0.8) |
| 2018–2021 | 12.4* (6.8 to 18.3) |
| 2021–2024 | -3.2* (-5.5 to -0.9) |
| **West** | |
| 2000-2003 | 2.1 (-0.1 to 4.2) |
| 2003-2018 | -0.2 (-0.5 to 0.2) |
| 2018–2021 | 8.7* (3.4 to 14.2) |
| 2021–2024 | -3.1* (-5.3 to -0.8) |
| **CAD Subgroup** | |
| **Chronic Ischemic Heart Disease** | |
| 2000-2018 | 0.7* (0.5 to 0.9) |
| 2018-2021 | 10.2* (5.2 to 15.4) |
| 2021-2024 | -2.8* (-4.9 to -0.6) |
| **Acute MI and Subsequent MI** | |
| 2000-2012 | -2.1* (-2.4 to -1.8) |
| 2012-2018 | -0.4 (-1.5 to 0.8) |
| 2018-2021 | 8.1* (3.1 to 13.4) |
| 2021-2024 | -7.5* (-9.6 to -5.4) |
| **Other Acute Ischemic Heart Disease** | |
| 2000-2003 | -2.8 (-13.1 to 8.8) |
| 2003-2006 | 17.6 (-4.9 to 45.5) |
| 2006-2018 | 0.8 (-0.1 to 1.7) |
| 2018-2021 | 8.1 (-4.4 to 22.3) |
| 2021-2024 | -2.6 (-7.9 to 3.0]) |
| **Angina Pectoris** | |
| 2000-2011 | -10.3* (-11.8 to -8.9) |
| 2011-2021 | 9.7* (7.5 to 11.9) |
| 2021-2024 | -2.9 (-9.6 to 4.3) |
| **HTN Subgroup** | |
| **Essential Hypertension** | |
| 2000-2008 | 0.9 (-0.3 to 2.0) |
| 2008-2018 | -2.1* (-2.9 to -1.2) |
| 2018-2021 | 9.0 (-0.5 to 19.5) |
| 2021-2024 | -6.1* (-10.3 to -1.8) |
| **Hypertensive Heart Disease** | |
| 2000-2002 | -2.6 (-7.7 to 2.8) |
| 2002-2012 | 3.2* (2.7 to 3.7) |
| 2006-2018 | 6.2* (5.3 to 7.2) |
| 2019-2021 | 12.4* (8.8 to 16.2) |
| 2021-2024 | 1.5 (-0.002 to 2.9) |
| **Hypertensive Renal Disease** | |
| 2000-2024 | 8.8* (6.8 to 10.8) |
| **Hypertensive Heart and Renal Disease** | |
| 2000-2024 | 2.9* (-4.8 to -0.9) |
| 2000-2024 | 16.9* (15.5 to 18.3) |
|  | |
| *CAD = Coronary Artery Disease; HTN = Hypertension;*  *NH: Non-Hispanic; APC: Annual Percent Change*  ** Indicates that Annual Percentage Change (APC) is significant (p value < 0.05)* | |

**Supplemental Table 2.** Annual percent change (APC) of coronary artery disease and hypertension-related age-adjusted mortality rates (per 100,000) among adults aged 25 and above in the United States, 2000 to 2024.

| **Age-Adjusted Rate /100,000 (95% CI)** | | | |
| --- | --- | --- | --- |
| **Year** | **Overall** | **Male** | **Female** |
| 2000 | 57.2 (56.9 – 57.6) | 66.2 (65.6 – 66.8) | 49.4 (49.0 – 49.8) |
| 2001 | 57.8 (57.4 – 58.1) | 67.4 (66.8 – 68.0) | 49.5 (49.1 – 49.9) |
| 2002 | 59.4 (59.1 – 59.8) | 69.8 (69.2 – 70.4) | 50.7 (50.3 – 51.1) |
| 2003 | 60.6 (60.2 – 60.9) | 72.5 (71.8 – 73.1) | 50.8 (50.4 – 51.2) |
| 2004 | 60.2 (59.9 – 60.6) | 72.8 (72.1 – 73.4) | 50.0 (49.5 – 50.4) |
| 2005 | 62.5 (62.2 – 62.9) | 75.9 (75.3 – 76.5) | 51.6 (51.2 – 52.0) |
| 2006 | 61.9 (61.6 – 62.3) | 76.8 (76.2 – 77.5) | 50.0 (49.6 – 50.4) |
| 2007 | 61.7 (61.3 – 62.0) | 76.8 (76.2 – 77.4) | 49.4 (49.0 – 49.8) |
| 2008 | 62.0 (61.6 – 62.3) | 77.8 (77.2 – 78.4) | 49.3 (48.9 – 49.7) |
| 2009 | 60.1 (59.8 – 60.5) | 76.5 (76.0 – 77.1) | 47.0 (46.6 – 47.4) |
| 2010 | 61.3 (61.0 – 61.6) | 78.6 (78.0 – 79.2) | 47.6 (47.2 – 48.0) |
| 2011 | 61.4 (61.0 – 61.7) | 79.4 (78.8 – 80.0) | 47.0 (46.6 – 47.4) |
| 2012 | 61.6 (61.3 – 61.9) | 80.3 (79.8 – 80.9) | 46.7 (46.3 – 47.0) |
| 2013 | 61.9 (61.5 – 62.2) | 81.3 (80.7 – 81.8) | 46.3 (45.9 – 46.6) |
| 2014 | 61.6 (61.3 – 61.9) | 82.1 (81.5 – 82.7) | 45.2 (44.8 – 45.5) |
| 2015 | 61.7 (61.3 – 62.0) | 82.5 (82.0 – 83.1) | 44.9 (44.5 – 45.2) |
| 2016 | 62.3 (62.0 – 62.6) | 83.6 (83.0 – 84.2) | 45.1 (44.8 – 45.5) |
| 2017 | 64.0 (63.6 – 64.3) | 86.5 (85.9 – 87.0) | 45.8 (45.5 – 46.2) |
| 2018 | 65.1 (64.8 – 65.5) | 89.0 (88.4 – 89.5) | 45.8 (45.5 – 46.1) |
| 2019 | 66.8 (66.5 – 67.1) | 91.3 (90.8 – 91.9) | 47.0 (46.6 – 47.3) |
| 2020 | 80.6 (80.3 – 81.0) | 111.0 (110.4 – 111.6) | 56.1 (55.7 – 56.5) |
| 2021 | 84.2 (83.9 – 84.6) | 115.9 (115.2 – 116.5) | 58.5 (58.1 – 58.9) |
| 2022 | 81.0 (80.7 – 81.3) | 112.5 (111.9 – 113.1) | 55.7 (55.4 – 56.1) |
| 2023 | 78.2 (77.8 – 78.5) | 107.9 (107.3 – 108.5) | 54.0 (53.6 – 54.3) |
| 2024 | 77.0 (76.6 – 77.3) | 106.4 (105.8 – 107.0) | 52.7 (52.3 – 53.0) |
| **Total** | 65.8 (65.7 – 65.8) | 85.9 (85.8 – 86.0) | 49.5 (49.4 – 49.6) |

**Supplemental Table 3.** Overall and sex-stratified coronary artery disease and hypertension-related age-adjusted mortality rates (per 100,000) among adults aged 25 and above in the United States, 2000 to 2024.

| Age-Adjusted Rate /100,000 (95% CI) | | | | | |
| --- | --- | --- | --- | --- | --- |
| Year | **NH American Indian or Alaska Native** | **NH Asian or Pacific Islander** | **NH Black or African American** | **NH White** | **Hispanics or Latino** |
| 2000 | 48.1 (43.0 – 53.3) | 46.6 (44.4 – 48.8) | 104.6 (102.9 – 106.2) | 52.7 (52.4 – 53.1) | 52.9 (51.2 – 54.5) |
| 2001 | 43.1 (38.2 – 47.9) | 44.1 (42.1 – 46.2) | 104.6 (103.0 – 106.3) | 53.2 (52.8 – 53.6) | 56.1 (54.5 – 57.7) |
| 2002 | 53.6 (48.2 – 58.9) | 45.3 (43.3 – 47.4) | 105.3 (103.7 – 107.0) | 55.1 (54.8 – 55.5) | 55.0 (53.5 – 56.6) |
| 2003 | 61.4 (55.7 – 67.1) | 45.5 (43.5 – 47.4) | 104.5 (102.9 – 106.1) | 56.5 (56.1 – 56.9) | 56.5 (55.0 – 58.0) |
| 2004 | 56.0 (50.6 – 61.4) | 45.3 (43.4 – 47.2) | 101.8 (100.2 – 103.4) | 56.5 (56.1 – 56.9) | 54.9 (53.4 – 56.4) |
| 2005 | 61.6 (56.1 – 67.2) | 45.3 (43.4 – 47.1) | 103.7 (102.1 – 105.3) | 58.7 (58.4 – 59.1) | 60.0 (58.5 – 61.5) |
| 2006 | 66.8 (61.2 – 72.5) | 45.6 (43.8 – 47.4) | 101.7 (100.2 – 103.2) | 58.4 (58.0 – 58.8) | 58.2 (56.8 – 59.7) |
| 2007 | 64.9 (59.4 – 70.4) | 44.0 (42.3 – 45.7) | 100.0 (98.5 – 101.5) | 58.6 (58.2 – 59.0) | 54.8 (53.4 – 56.1) |
| 2008 | 58.9 (53.9 – 64.0) | 43.6 (42.0 – 45.3) | 97.2 (95.7 – 98.6) | 59.3 (58.9 – 59.7) | 54.9 (53.6 – 56.2) |
| 2009 | 65.8 (60.5 – 71.1) | 43.1 (41.5 – 44.6) | 91.7 (90.3 – 93.1) | 57.7 (57.4 – 58.1) | 52.3 (51.1 – 53.5) |
| 2010 | 65.5 (60.3 – 70.6) | 43.7 (42.1 – 45.2) | 90.7 (89.3 – 92.1) | 59.1 (58.8 – 59.5) | 55.1 (53.9 – 56.4) |
| 2011 | 69.2 (64.0 – 74.3) | 41.5 (40.0 – 42.9) | 89.6 (88.2 – 90.9) | 59.6 (59.2 – 60.0) | 53.4 (52.2 – 54.5) |
| 2012 | 70.2 (65.2 – 75.2) | 41.1 (39.7 – 42.5) | 88.3 (87.0 – 89.7) | 60.1 (59.7 – 60.4) | 52.7 (51.5 – 53.8) |
| 2013 | 67.5 (62.7 – 72.2) | 41.4 (40.0 – 42.8) | 88.4 (87.1 – 89.7) | 60.2 (59.8 – 60.6) | 54.6 (53.5 – 55.7) |
| 2014 | 72.3 (67.5 – 77.1) | 38.3 (37.0 – 39.5) | 85.0 (83.8 – 86.3) | 60.5 (60.2 – 60.9) | 53.2 (52.1 – 54.3) |
| 2015 | 68.8 (64.3 – 73.3) | 38.0 (36.8 – 39.2) | 84.5 (83.3 – 85.7) | 60.7 (60.4 – 61.1) | 52.1 (51.1 – 53.1) |
| 2016 | 73.7 (69.1 – 78.3) | 37.9 (36.7 – 39.0) | 86.3 (85.0 – 87.5) | 61.4 (61.0 – 61.7) | 52.8 (51.8 – 53.8) |
| 2017 | 74.4 (69.9 – 79.0) | 39.2 (38.0 – 40.3) | 85.9 (84.7 – 87.1) | 63.5 (63.1 – 63.8) | 53.1 (52.2 – 54.1) |
| 2018 | 71.0 (66.8 – 75.3) | 39.1 (38.0 – 40.3) | 87.4 (86.2 – 88.5) | 64.9 (64.6 – 65.3) | 52.8 (51.8 – 53.8) |
| 2019 | 68.9 (64.8 – 73.1) | 39.1 (38.0 – 40.2) | 89.0 (87.8 – 90.2) | 66.8 (66.5 – 67.2) | 55.2 (54.2 – 56.1) |
| 2020 | 83.2 (78.8 – 87.7) | 49.0 (47.8 – 50.1) | 115.7 (114.3 – 117.0) | 78.3 (77.9 – 78.7) | 76.6 (75.5 – 77.7) |
| 2021 | 85.1 (80.5 – 89.9) | 49.1 (47.9 – 50.3) | 113.2 (111.8 – 114.5) | 84.7 (84.2 – 85.1) | 70.5 (69.4 – 71.5) |
| 2022 | 79.2 (74.8 – 83.7) | 44.4 (43.4 – 45.6) | 106.8 (105.5 – 108.1) | 82.3 (81.9 – 82.7) | 65.1 (64.1 – 66.1) |
| 2023 | 83.4 (79.1 – 88.0) | 42.0 (40.9 – 43.0) | 101.4 (100.2 – 102.7) | 79.9 (79.5 – 80.3) | 61.2 (60.3 – 62.2) |
| 2024 | 77.6 (73.5 – 81.9) | 41.7 (40.7 – 42.7) | 99.5 (98.3 – 100.7) | 79.1 (78.7 – 79.5) | 58.3 (57.4 – 59.2) |
| Total | 68.4 (67.4 – 69.4) | 42.7 (42.4 – 43.0) | 96.9 (96.6 – 97.2) | 63.7 (63.6 – 63.8) | 57.4 (57.2 – 56.6) |

*NH: Non-Hispanic*

**Supplemental Table 4.** Race/ Ethnicity-stratified coronary artery disease and hypertension-related age-adjusted mortality rates (per 100,000) among adults aged 25 and above in the United States, 2000 to 2024.

| Age-Adjusted Rate /100,000 (95% CI) | | |
| --- | --- | --- |
| Year | **Metropolitan** | **Non-Metropolitan** |
| 2000 | 57.7 (57.3 – 58.1) | 55.1 (54.3 – 55.9) |
| 2001 | 58.3 (57.9 – 58.7) | 55.3 (54.6 – 56.1) |
| 2002 | 59.4 (59.0 – 59.8) | 59.6 (58.8 – 60.4) |
| 2003 | 60.2 (59.8 – 60.5) | 62.5 (61.7 – 63.3) |
| 2004 | 59.8 (59.4 – 60.2) | 62.3 (61.5 – 63.1) |
| 2005 | 61.9 (61.5 – 62.3) | 65.6 (64.8 – 66.4) |
| 2006 | 61.6 (61.2 – 62.0) | 64.0 (63.2 – 64.8) |
| 2007 | 61.0 (60.6 – 61.3) | 65.0 (64.2 – 65.8) |
| 2008 | 61.0 (60.7 – 61.4) | 66.4 (65.5 – 67.2) |
| 2009 | 58.9 (58.6 – 59.3) | 65.6 (64.8 – 66.5) |
| 2010 | 60.3 (60.0 – 60.7) | 66.2 (65.3 – 67.0) |
| 2011 | 60.6 (60.2 – 60.9) | 65.7 (64.9 – 66.5) |
| 2012 | 60.4 (60.0 – 60.8) | 67.9 (67.0 – 68.7) |
| 2013 | 60.6 (60.3 – 61.0) | 68.0 (67.2 – 68.8) |
| 2014 | 60.0 (59.7 – 60.4) | 69.7 (68.9 – 70.5) |
| 2015 | 59.8 (59.4 – 60.1) | 71.5 (70.6 – 72.3) |
| 2016 | 60.5 (60.2 – 60.9) | 71.3 (70.5 – 72.1) |
| 2017 | 62.1 (61.7 – 62.4) | 73.8 (73.0 – 74.7) |
| 2018 | 63.0 (62.7 – 63.4) | 76.1 (75.2 – 76.9) |
| 2019 | 64.3 (64.0 – 64.7) | 79.9 (79.0 – 80.7) |
| 2020 | 77.8 (77.4 – 78.2) | 95.8 (94.9 – 96.7) |
| Total | 61.6 (61.5 – 61.6) | 68.2 (68.0 – 68.3) |

**Supplemental Table 5.** Urbanization-stratified coronary artery disease and hypertension-related age-adjusted mortality rates (per 100,000) among adults aged 25 and above in the United States, 2000 to 2020.

| Age-Adjusted Rate /100,000 (95% CI) | | | | |
| --- | --- | --- | --- | --- |
| Year | **Northeast** | **Midwest** | **South** | **West** |
| 2000 | 57.9 (57.1 – 58.7) | 57.2 (56.5 – 57.9) | 57.6 (57.0 – 58.1) | 56.0 (55.2 – 56.7) |
| 2001 | 57.9 (57.2 – 58.7) | 57.0 (56.3 – 57.8) | 59.0 (58.4 – 59.6) | 56.2 (55.4 – 56.9) |
| 2002 | 59.4 (58.6 – 60.2) | 58.7 (58.0 – 59.4) | 60.3 (59.7 – 60.9) | 58.6 (57.8 – 59.4) |
| 2003 | 59.0 (58.2 – 59.7) | 59.6 (58.9 – 60.3) | 62.6 (62.0 – 63.2) | 59.7 (58.9 – 60.4) |
| 2004 | 59.3 (58.5 – 60.0) | 59.3 (58.6 – 60.0) | 61.4 (60.8 – 62.0) | 59.9 (59.1 – 60.6) |
| 2005 | 60.7 (59.9 – 61.5) | 61.1 (60.4 – 61.9) | 65.2 (64.6 – 65.8) | 61.2 (60.4 – 61.9) |
| 2006 | 57.8 (57.1 – 58.5) | 60.6 (59.9 – 61.3) | 64.9 (64.3 – 65.5) | 62.4 (61.6 – 63.1) |
| 2007 | 58.2 (57.5 – 59.0) | 61.5 (60.8 – 62.3) | 64.3 (63.7 – 64.9) | 60.2 (59.5 – 60.9) |
| 2008 | 58.0 (57.3 – 58.8) | 63.4 (62.7 – 64.1) | 63.5 (63.0 – 64.1) | 61.2 (60.4 – 61.9) |
| 2009 | 56.6 (55.9 – 57.4) | 59.6 (58.9 – 60.2) | 63.1 (62.5 – 63.7) | 58.7 (58.0 – 59.4) |
| 2010 | 59.9 (59.2 – 60.7) | 60.5 (59.8 – 61.1) | 63.9 (63.4 – 64.5) | 58.8 (58.1 – 59.5) |
| 2011 | 59.8 (59.1 – 60.5) | 61.5 (60.9 – 62.2) | 63.0 (62.4 – 63.5) | 59.5 (58.9 – 60.2) |
| 2012 | 59.6 (58.9 – 60.3) | 61.4 (60.7 – 62.1) | 64.2 (63.6 – 64.7) | 59.1 (58.4 – 59.8) |
| 2013 | 59.8 (59.0 – 60.5) | 60.7 (60.0 – 61.4) | 64.8 (64.2 – 65.3) | 59.6 (58.9 – 60.3) |
| 2014 | 58.5 (57.8 – 59.2) | 61.6 (61.0 – 62.3) | 65.1 (64.6 – 65.7) | 58.1 (57.5 – 58.8) |
| 2015 | 58.4 (57.7 – 59.1) | 62.4 (61.8 – 63.1) | 64.8 (64.3 – 65.4) | 58.1 (57.5 – 58.8) |
| 2016 | 58.6 (57.9 – 59.3) | 61.7 (61.0 – 62.4) | 65.8 (65.3 – 66.3) | 59.9 (59.2 – 60.6) |
| 2017 | 59.0 (58.3 – 59.7) | 64.3 (63.6 – 65.0) | 67.8 (67.3 – 68.3) | 61.2 (60.5 – 61.8) |
| 2018 | 62.0 (61.2 – 62.7) | 64.9 (64.2 – 65.5) | 69.0 (68.4 – 69.5) | 61.3 (60.6 – 61.9) |
| 2019 | 62.6 (61.9 – 63.3) | 65.8 (65.2 – 66.5) | 72.5 (71.9 – 73.0) | 61.8 (61.1 – 62.4) |
| 2020 | 76.7 (75.9 – 77.5) | 80.2 (79.5 – 81.0) | 88.3 (87.7 – 88.9) | 71.1 (70.4 – 71.8) |
| 2021 | 72.3 (71.5 – 73.0) | 82.7 (81.9 – 83.4) | 95.3 (94.7 – 95.9) | 76.9 (76.2 – 77.7) |
| 2022 | 71.2 (70.5 – 72.0) | 79.8 (79.1 – 80.6) | 91.1 (90.5 – 91.7) | 73.0 (72.3 – 73.7) |
| 2023 | 67.5 (66.7 – 68.2) | 76.0 (75.3 – 76.7) | 88.9 (88.3 – 89.5) | 70.8 (70.2 – 71.5) |
| 2024 | 65.1 (64.4 – 65.8) | 75.9 (75.2 – 76.6) | 87.4 (86.8 – 88.0) | 69.9 (69.2 – 70.5) |
| Total | 61.6 (61.4 – 61.7) | 65.0 (64.9 – 65.1) | 70.3 (71.0 – 71.4) | 62.5 (62.4 – 62.7) |

**Supplemental Table 6.** Census Region-stratified coronary artery disease and hypertension-related age-adjusted mortality rates (per 100,000) among adults aged 25 and above in the United States, 2000 to 2024.

| **State** | **Age-Adjusted Rate /100,000 (95% CI)** | **Percentile (%)** |
| --- | --- | --- |
| Utah | 35.5 (34.9 – 36.1) | 0 |
| Massachusetts | 40.4 (40.1 – 40.8) | 2 |
| Connecticut | 44.4 (44.0 – 44.9) | 4 |
| Alabama | 46.3 (45.8 – 46.7) | 6 |
| Maine | 46.8 (46.0 – 47.6) | 8 |
| Alaska | 47.2 (45.6 – 48.7) | 10 |
| Kansas | 48.3 (47.7 – 48.9) | 12 |
| Virginia | 48.7 (48.4 – 49.1) | 14 |
| Georgia | 49.2 (48.8 – 49.6) | 16 |
| Colorado | 49.9 (49.4 – 50.4) | 18 |
| Illinois | 50.2 (49.9 – 50.4) | 20 |
| Hawaii | 51.7 (50.9 – 52.6) | 22 |
| New Hampshire | 52.1 (51.2 – 53.0) | 24 |
| Montana | 52.5 (51.5 – 53.5) | 26 |
| Idaho | 52.6 (51.7 – 53.5) | 28 |
| Oregon | 53.5 (52.9 – 54.0) | 30 |
| Nebraska | 55.5 (54.8 – 56.3) | 32 |
| New Jersey | 57.3 (56.9 – 57.7) | 34 |
| Wyoming | 57.5 (56.0 – 59.0) | 36 |
| Florida | 58.6 (58.4 – 58.8) | 38 |
| Arizona | 58.6 (58.2 – 59.1) | 40 |
| Pennsylvania | 58.8 (58.5 – 59.1) | 42 |
| Missouri | 59.0 (58.6 – 59.5) | 44 |
| Minnesota | 60.4 (60.0 – 60.9) | 46 |
| Washington | 61.1 (60.6 – 61.5) | 48 |
| South Dakota | 64.5 (63.3 – 65.8) | 50 |
| Indiana | 64.7 (64.2 – 65.1) | 52 |
| Wisconsin | 65.5 (65.0 – 66.0) | 54 |
| North Carolina | 66.0 (65.6 – 66.4) | 56 |
| Iowa | 67.3 (66.7 – 67.9) | 58 |
| North Dakota | 67.9 (66.5 – 69.2) | 60 |
| California | 68.0 (67.8 – 68.2) | 62 |
| Nevada | 69.5 (68.8 – 70.3) | 64 |
| Michigan | 70.6 (70.2 – 71.0) | 66 |
| Kentucky | 70.8 (70.2 – 71.3) | 68 |
| Maryland | 70.9 (70.4 – 71.5) | 70 |
| Louisiana | 71.9 (71.3 – 72.5) | 72 |
| South Carolina | 74.2 (73.6 – 74.8) | 74 |
| New York | 75.3 (75.1 – 75.6) | 76 |
| Delaware | 79.3 (77.9 – 80.6) | 78 |
| New Mexico | 80.6 (79.7 – 81.6) | 80 |
| Texas | 84.8 (84.5 – 85.1) | 82 |
| Ohio | 85.6 (85.2 – 85.9) | 84 |
| Arkansas | 86.6 (85.8 – 87.4) | 86 |
| Tennessee | 89.2 (88.7 – 89.8) | 88 |
| Rhode Island | 89.7 (88.4 – 90.9) | 90 |
| West Virginia | 91.7 (90.7 – 92.7) | 92 |
| Vermont | 99.0 (97.2 – 100.7) | 94 |
| Oklahoma | 102.6 (101.9 – 103.4) | 96 |
| Mississippi | 107.8 (106.9 – 108.7) | 98 |
| District of Columbia | 121.9 (119.7 – 124.1) | 100 |

**Supplemental Table 7.** State-stratified coronary artery disease and hypertension-related age-adjusted mortality rates (per 100,000) among adults aged 25 and above in the United States, 2000 to 2024.

| Age-Adjusted Rate /100,000 (95% CI) | | | |
| --- | --- | --- | --- |
| Year | **Young-Aged (25-44 years)** | **Middle-Aged (45-64 years)** | **Advanced-Aged (65+ years)** |
| 2000 | 2.0 (1.9 – 2.1) | 25.6 (25.2 – 26.0) | 243.1 (241.4 – 244.7) |
| 2001 | 2.0 (1.9 – 2.1) | 25.5 (25.1 – 25.9) | 246.0 (244.3 – 247.6) |
| 2002 | 2.1 (2.0 – 2.2) | 25.6 (25.2 – 25.9) | 254.3 (252.6 – 255.9) |
| 2003 | 2.4 (2.2 – 2.5) | 26.7 (26.3 – 27.1) | 257.4 (255.8 – 259.1) |
| 2004 | 2.4 (2.3 – 2.5) | 26.8 (26.5 – 27.2) | 255.3 (253.6 – 256.9) |
| 2005 | 2.6 (2.5 – 2.7) | 28.4 (28.0 – 28.8) | 264.0 (262.4 – 265.7) |
| 2006 | 2.7 (2.6 – 2.8) | 29.1 (28.7 – 29.5) | 259.5 (257.9 – 261.1) |
| 2007 | 2.8 (2.7 – 2.9) | 29.1 (28.7 – 29.5) | 257.9 (256.3 – 259.5) |
| 2008 | 2.8 (2.7 – 3.0) | 29.7 (29.3 – 30.1) | 258.2 (256.7 – 259.8) |
| 2009 | 2.8 (2.7 – 2.9) | 29.3 (29.0 – 29.7) | 249.6 (248.0 – 251.1) |
| 2010 | 2.8 (2.7 – 2.9) | 30.0 (29.7 – 30.4) | 254.3 (252.7 – 255.8) |
| 2011 | 2.9 (2.8 – 3.1) | 31.0 (30.6 – 31.4) | 252.7 (251.2 – 254.2) |
| 2012 | 3.1 (3.0 – 3.2) | 31.5 (31.2 – 31.9) | 252.5 (251.0 – 254.0) |
| 2013 | 3.2 (3.1 – 3.3) | 32.2 (31.8 – 32.6) | 252.4 (250.9 – 253.9) |
| 2014 | 3.3 (3.2 – 3.4) | 33.0 (32.7 – 33.4) | 249.5 (248.0 – 250.9) |
| 2015 | 3.3 (3.2 – 3.4) | 33.3 (32.9 – 33.7) | 249.1 (247.7 – 250.6) |
| 2016 | 3.5 (3.3 – 3.6) | 34.8 (34.4 – 35.2) | 249.3 (247.9 – 250.8) |
| 2017 | 3.6 (3.4 – 3.7) | 35.5 (35.1 – 35.9) | 256.5 (255.1 – 257.9) |
| 2018 | 3.6 (3.4 – 3.7) | 36.8 (36.4 – 37.2) | 260.2 (258.8 – 261.6) |
| 2019 | 3.7 (3.6 – 3.9) | 37.2 (36.8 – 37.6) | 267.8 (266.4 – 269.3) |
| 2020 | 4.4 (4.2 – 4.5) | 44.2 (43.8 – 44.7) | 324.5 (323.0 – 326.1) |
| 2021 | 4.6 (4.4 – 4.7) | 45.9 (45.5 – 46.4) | 339.4 (337.8 – 341.0) |
| 2022 | 4.6 (4.4 – 4.7) | 44.1 (43.7 – 44.6) | 326.0 (324.5 – 327.5) |
| 2023 | 4.4 (4.3 – 4.6) | 42.5 (42.1 – 43.0) | 314.9 (313.4 – 316.4) |
| 2024 | 4.5 (4.4 – 4.7) | 41.6 (41.2 – 42.1) | 309.9 (308.4 – 311.3) |
| Total | 3.2 (3.2 – 3.2) | 33.6 (33.5 – 33.6) | 272.1 (271.8 – 272.5) |

**Supplemental Table 8.** Age group-stratified coronary artery disease and hypertension-related crude mortality rates (per 100,000) among adults aged 25 and above in the United States, 2000 to 2024.

| Age-Adjusted Rate /100,000 (95% CI) | | | | |
| --- | --- | --- | --- | --- |
| Year | **Angina Pectoris** | **Acute MI and Subsequent MI** | **Other Acute Ischemic Heart Diseases** | **Chronic Ischemic Heart Disease** |
| 2000 | 0.5 (0.4 – 0.5) | 19.8 (19.6 – 20.0) | 0.7 (0.6 – 0.7) | 46.2 (45.9 – 46.5) |
| 2001 | 0.4 (0.4 – 0.5) | 19.4 (19.2 – 19.6) | 0.7 (0.6 – 0.7) | 46.9 (46.6 – 47.2) |
| 2002 | 0.4 (0.4 – 0.4) | 19.7 (19.5 – 19.9) | 0.7 (0.6 – 0.7) | 48.4 (48.0 – 48.7) |
| 2003 | 0.3 (0.3 – 0.3) | 19.4 (19.2 – 19.6) | 0.6 (0.6 – 0.7) | 49.5 (49.2 – 49.8) |
| 2004 | 0.3 (0.3 – 0.3) | 18.7 (18.5 – 18.9) | 0.6 (0.5 – 0.6) | 49.7 (49.4 – 50.0) |
| 2005 | 0.2 (0.2 – 0.3) | 18.7 (18.5 – 18.9) | 0.9 (0.8 – 0.9) | 51.7 (51.4 – 52.0) |
| 2006 | 0.2 (0.2 – 0.2) | 18.0 (17.8 – 18.2) | 1.0 (0.9 – 1.0) | 51.4 (51.0 – 51.7) |
| 2007 | 0.2 (0.2 – 0.2) | 17.2 (17.0 – 17.4) | 1.1 (1.0 – 1.1) | 51.5 (51.1 – 51.8) |
| 2008 | 0.2 (0.2 – 0.2) | 17.5 (17.3 – 17.7) | 1.1 (1.0 – 1.1) | 51.5 (51.2 – 51.8) |
| 2009 | 0.2 (0.2 – 0.2) | 16.4 (16.2 – 16.5) | 1.0 (0.9 – 1.0) | 50.2 (49.9 – 50.5) |
| 2010 | 0.2 (0.1 – 0.2) | 16.1 (16.0 – 16.3) | 1.0 (1.0 – 1.1) | 51.6 (51.3 – 51.9) |
| 2011 | 0.1 (0.1 – 0.2) | 16.0 (15.8 – 16.1) | 1.0 (0.9 – 1.0) | 51.8 (51.5 – 52.1) |
| 2012 | 0.2 (0.1 – 0.2) | 15.8 (15.7 – 16.0) | 0.9 (0.9 – 1.0) | 52.0 (51.7 – 52.3) |
| 2013 | 0.2 (0.1 – 0.2) | 15.7 (15.6 – 15.9) | 1.0 (1.0 – 1.1) | 52.3 (52.0 – 52.6) |
| 2014 | 0.2 (0.2 – 0.2) | 15.6 (15.4 – 15.7) | 1.1 (1.1 – 1.1) | 52.1 (51.8 – 52.4) |
| 2015 | 0.2 (0.2 – 0.2) | 15.6 (15.4 – 15.7) | 1.1 (1.1 – 1.1) | 52.1 (51.8 – 52.4) |
| 2016 | 0.2 (0.2 – 0.2) | 15.4 (15.3 – 15.6) | 1.1 (1.0 – 1.1) | 52.7 (52.4 – 53.0) |
| 2017 | 0.3 (0.2 – 0.3) | 15.5 (15.3 – 15.7) | 1.1 (1.1 – 1.2) | 54.2 (53.9 – 54.5) |
| 2018 | 0.3 (0.2 – 0.3) | 15.8 (15.6 – 15.9) | 1.1 (1.1 – 1.1) | 55.3 (55.0 – 55.5) |
| 2019 | 0.3 (0.3 – 0.3) | 15.8 (15.6 – 15.9) | 1.1 (1.1 – 1.2) | 57.0 (56.8 – 57.3) |
| 2020 | 0.3 (0.3 – 0.3) | 18.4 (18.3 – 18.6) | 1.3 (1.2 – 1.3) | 69.0 (68.7 – 69.3) |
| 2021 | 0.3 (0.3 – 0.4) | 19.4 (19.3 – 19.6) | 1.4 (1.3 – 1.4) | 71.8 (71.5 – 72.1) |
| 2022 | 0.4 (0.3 – 0.4) | 18.0 (17.9 – 18.2) | 1.4 (1.3 – 1.4) | 69.6 (69.3 – 69.9) |
| 2023 | 0.3 (0.3 – 0.3) | 16.3 (16.1 – 16.4) | 1.3 (1.3 – 1.3) | 68.0 (67.7 – 68.3) |
| 2024 | 0.3 (0.3 – 0.3) | 15.6 (15.4 – 15.7) | 1.3 (1.2 – 1.3) | 67.3 (67.0 – 67.6) |
| Total | 0.3 (0.3 – 0.3) | 17.1 (17.1 – 17.1) | 1.0 (1.0 – 1.0) | 55.4 (55.4 – 55.5) |

*MI: Myocardial Infarction*

**Supplemental Table 9.** Coronary artery disease subgroups-stratified coronary artery disease and hypertension-related age-adjusted mortality rates (per 100,000) among adults aged 25 and above in the United States, 2000 to 2024.

| Age-Adjusted Rate /100,000 (95% CI) | | | | |
| --- | --- | --- | --- | --- |
| Year | **Essential Hypertension** | **Hypertensive Heart Disease** | **Hypertensive Renal Disease** | **Hypertensive Heart and Renal Disease** |
| 2000 | 47.9 (47.5 – 48.2) | 7.5 (7.4 – 7.6) | 1.7 (1.7 – 1.8) | 0.6 (0.5 – 0.6) |
| 2001 | 48.4 (48.0 – 48.7) | 7.5 (7.4 – 7.6) | 1.8 (1.7 – 1.8) | 0.5 (0.5 – 0.5) |
| 2002 | 50.5 (50.1 – 50.8) | 7.0 (6.9 – 7.1) | 1.9 (1.8 – 1.9) | 0.5 (0.5 – 0.5) |
| 2003 | 51.1 (50.8 – 51.4) | 7.5 (7.3 – 7.6) | 1.9 (1.9 – 2.0) | 0.5 (0.5 – 0.5) |
| 2004 | 50.7 (50.4 – 51.0) | 7.4 (7.3 – 7.6) | 1.9 (1.8 – 2.0) | 0.5 (0.5 – 0.5) |
| 2005 | 52.5 (52.2 – 52.8) | 7.9 (7.8 – 8.1) | 1.9 (1.9 – 2.0) | 0.5 (0.5 – 0.5) |
| 2006 | 51.5 (51.1 – 51.8) | 8.5 (8.4 – 8.6) | 2.0 (1.9 – 2.0) | 0.5 (0.4 – 0.5) |
| 2007 | 51.0 (50.7 – 51.4) | 8.6 (8.4 – 8.7) | 2.0 (1.9 – 2.0) | 0.5 (0.5 – 0.5) |
| 2008 | 51.3 (51.0 – 51.6) | 8.7 (8.6 – 8.8) | 1.9 (1.9 – 2.0) | 0.5 (0.4 – 0.5) |
| 2009 | 49.2 (48.9 – 49.5) | 9.0 (8.8 – 9.1) | 1.9 (1.9 – 2.0) | 0.4 (0.4 – 0.5) |
| 2010 | 50.0 (49.7 – 50.3) | 9.3 (9.2 – 9.4) | 1.9 (1.9 – 2.0) | 0.4 (0.4 – 0.5) |
| 2011 | 51.7 (51.4 – 52.0) | 9.5 (9.3 – 9.6) | 0.2 (0.2 – 0.3) | 0.3 (0.3 – 0.4) |
| 2012 | 51.5 (51.1 – 51.8) | 9.8 (9.7 – 10.0) | 0.3 (0.3 – 0.3) | 0.4 (0.4 – 0.4) |
| 2013 | 45.7 (45.4 – 46.0) | 10.4 (10.2 – 10.5) | 5.7 (5.6 – 5.8) | 0.5 (0.5 – 0.5) |
| 2014 | 44.6 (44.3 – 44.9) | 11.2 (11.0 – 11.3) | 5.8 (5.7 – 5.9) | 0.6 (0.5 – 0.6) |
| 2015 | 43.8 (43.5 – 44.1) | 11.7 (11.6 – 11.8) | 6.0 (6.0 – 6.2) | 0.6 (0.6 – 0.6) |
| 2016 | 43.3 (43.0 – 43.5) | 12.6 (12.5 – 12.8) | 6.2 (6.1 – 6.3) | 0.7 (0.7 – 0.7) |
| 2017 | 43.9 (43.6 – 44.1) | 13.3 (13.2 – 13.4) | 6.5 (6.4 – 6.6) | 0.8 (0.8 – 0.8) |
| 2018 | 44.0 (43.7 – 44.2) | 14.2 (14.1 – 14.3) | 6.7 (6.6 – 6.8) | 0.9 (0.8 – 0.9) |
| 2019 | 43.9 (43.6 – 44.1) | 15.5 (15.3 – 15.7) | 7.2 (7.0 – 7.2) | 1.0 (1.0 – 1.1) |
| 2020 | 52.4 (52.1 – 52.7) | 19.0 (18.9 – 19.2) | 8.7 (8.6 – 8.8) | 1.5 (1.4 – 1.5) |
| 2021 | 54.9 (54.6 – 55.1) | 19.8 (19.6 – 20.0) | 9.2 (9.1 – 9.3) | 1.6 (1.5 – 1.6) |
| 2022 | 51.1 (50.8 – 51.4) | 20.3 (20.1 – 20.5) | 8.8 (8.7 – 8.9) | 2.1 (2.0 – 2.1) |
| 2023 | 47.9 (47.6 – 48.1) | 20.5 (20.4 – 20.7) | 8.8 (8.6 – 8.9) | 2.2 (2.2 – 2.3) |
| 2024 | 46.0 (45.7 – 46.2) | 21.3 (21.1 – 21.4) | 8.6 (8.5 – 8.7) | 2.4 (2.3 – 2.4) |
| Total | 48.6 (48.6 – 48.7) | 12.2 (12.2 – 12.3) | 4.6 (4.6 – 4.6) | 0.9 (0.9 – 0.9) |

**Supplemental Table 10.** Hypertension subgroups-stratified coronary artery disease and hypertension-related age-adjusted mortality rates (per 100,000) among adults aged 25 and above in the United States, 2000 to 2024.

**Supplementary Figure 1.** Census region-stratified coronary artery disease and hypertension-related age-adjusted mortality rates (per 100,000) among adults aged 25 and above in the United States, 2000 to 2024.

**Supplementary Figure 2.** Coronary artery disease and hypertension-related age-adjusted mortality rates stratified by age groups 25 to 85+ years in the United States, 2000 to 2024.

**Supplementary Figure 3.** Percent total deaths of coronary artery disease and hypertension-related mortality stratified by place of death among adults aged 25 and above in the United States, 2000 to 2024.

**Supplementary Figure 4.** Trends in coronary artery disease and hypertension-related age-adjusted mortality rates (per 100,000) stratified by coronary artery disease subgroups among adults aged 25 and above in the United States, 2000 to 2024.

**Supplementary Figure 5.** Trends in coronary artery disease and hypertension-related age-adjusted mortality rates (per 100,000) stratified by hypertension subgroups among adults aged 25 and above in the United States, 2000 to 2024.
